# Gut Microbiome in Chronic Coronary Syndrome Patients

**DOI:** 10.3390/jcm10215074

**Published:** 2021-10-29

**Authors:** Emilia Sawicka-Smiarowska, Kinga Bondarczuk, Witold Bauer, Magdalena Niemira, Anna Szalkowska, Justyna Raczkowska, Miroslaw Kwasniewski, Ewa Tarasiuk, Marlena Dubatowka, Magda Lapinska, Malgorzata Szpakowicz, Zofia Stachurska, Anna Szpakowicz, Pawel Sowa, Andrzej Raczkowski, Marcin Kondraciuk, Magdalena Gierej, Joanna Motyka, Jacek Jamiolkowski, Mateusz Bondarczuk, Malgorzata Chlabicz, Jolanta Bucko, Marcin Kozuch, Slawomir Dobrzycki, Jerzy Bychowski, Wlodzimierz Jerzy Musial, Adrian Godlewski, Michal Ciborowski, Attila Gyenesei, Adam Kretowski, Karol Adam Kaminski

**Affiliations:** 1 Department of Population Medicine and Lifestyle Diseases Prevention, Medical University of Bialystok, 15-269 Bialystok, Poland; emiliasawickak@gmail.com (E.S.-S.); Marlena.paniczko@umb.edu.pl (M.D.); magda.lapinska@umb.edu.pl (M.L.); malgorzata.szpakowicz@umb.edu.pl (M.S.); zofia.stachurska@umb.edu.pl (Z.S.); mailtosowa@gmail.com (P.S.); andrzej.raczkowski@umb.edu.pl (A.R.); marcin.kondraciuk@umb.edu.pl (M.K.); gierejmagda@gmail.com (M.G.); joanna.motyka@umb.edu.pl (J.M.); jacek909@wp.pl (J.J.); malgorzata.chlabicz@umb.edu.pl (M.C.); 2 Department of Cardiology, Medical University of Bialystok, 15-269 Bialystok, Poland; ewa-tarasiuk@o2.pl (E.T.); akodzi@poczta.onet.pl (A.S.); musialwj@poczta.onet.pl (W.J.M.); 3 Centre for Bioinformatics and Data Analysis, Medical University of Bialystok, 15-269 Bialystok, Poland; kinga.bondarczuk@umb.edu.pl (K.B.); miroslaw.kwasniewski@umb.edu.pl (M.K.); mateusz.bondarczuk@umb.edu.pl (M.B.); 4 Clinical Research Centre, Medical University of Bialystok, 15-269 Bialystok, Poland; witold.bauer@umb.edu.pl (W.B.); magdalena.niemira@umb.edu.pl (M.N.); anna.szalkowska@umb.edu.pl (A.S.); justyna.raczkowska@umb.edu.pl (J.R.); cbk@umb.edu.pl (A.G.); michalciborowski79@gmail.com (M.C.); gyenesei.attila@pte.hu (A.G.); adamkretowski@wp.pl (A.K.); 5 Department of Invasive Cardiology, Medical University of Bialystok, 15-269 Bialystok, Poland; marcin.kozuch@umb.edu.pl (M.K.); slawek_dobrzycki@yahoo.com (S.D.); 6 Department of Cardiology, Bialystok Regional Hospital, 15-950 Bialystok, Poland; jolantabucko@wp.pl (J.B.); kardiologia@sniadecja.pl (J.B.)

**Keywords:** gut microbiome, coronary artery disease, *Firmicutes/Bacteroidetes* ratio, microbiome dysbiosis, targeted metabolomics, phosphatidylcholine

## Abstract

Despite knowledge of classical coronary artery disease (CAD) risk factors, the morbidity and mortality associated with this disease remain high. Therefore, new factors that may affect the development of CAD, such as the gut microbiome, are extensively investigated. This study aimed to evaluate gut microbiome composition in CAD patients in relation to the control group. We examined 169 CAD patients and 166 people in the control group, without CAD, matched in terms of age and sex to the study group. Both populations underwent a detailed health assessment. The microbiome analysis was based on the V3–V4 region of the 16S rRNA gene (NGS method). Among 4074 identified taxonomic units in the whole population, 1070 differed between study groups. The most common bacterial types were *Firmicutes*, *Bacteroidetes*, *Proteobacteria,* and *Actinobacteria*. Furthermore, a higher *Firmicutes/Bacteroidetes* ratio in the CAD group compared with the control was demonstrated. *Firmicutes/Bacteroidetes* ratio, independent of age, sex, CAD status, LDL cholesterol concentration, and statins treatment, was related to altered phosphatidylcholine concentrations obtained in targeted metabolomics. Altered alpha-biodiversity (Kruskal–Wallis test, *p* = 0.001) and beta-biodiversity (Bray–Curtis metric, *p* < 0.001) in the CAD group were observed. Moreover, a predicted functional analysis revealed some taxonomic units, metabolic pathways, and proteins that might be characteristic of the CAD patients’ microbiome, such as increased expressions of 6-phospho-β-glucosidase and protein-N(pi)-phosphohistidine-sugar phosphotransferase and decreased expressions of DNA topoisomerase, oxaloacetate decarboxylase, and 6-beta-glucosidase. In summary, CAD is associated with altered gut microbiome composition and function.

## 1. Introduction

Among the non-infectious diseases, cardiovascular pathologies are significant causes of mortality (49% of all deaths) [1]. Among them, coronary artery disease (CAD) is one of the most common causes of hospitalization (more than 20% of all hospitalizations), death, and disability in Poland and Europe [2]. Furthermore, CAD causes a decrease in quality of life and leads to very high social and economic costs, affecting both individuals and all of society.

It is well-known that the most common cause of CAD is atherosclerosis, which is tightly associated with a wide number of modifiable and non-modifiable risk factors [3]. Classical CAD risk factors were reliably described based on a Framingham cohort study [4]. Despite knowledge of these risk factors, morbidity and mortality associated with CAD remain high. As a consequence, it is necessary to seek new factors that may play a role in the development of CAD and influence the prognosis in this group of patients.

Recently, particular attention has been drawn to the endogenous microflora of the human body (microbiota), which inhabit various sites of the human body (with the largest amount in the intestines) and remains relatively unexplored. It should be emphasized that many bacterial species fail to grow in vitro. Therefore, new, innovative identification techniques (for instance, Next Generation Sequencing method—NGS—based on the V3–V4 region of the 16S rRNA) and new bioinformatics techniques are utilized. The 16S rRNA included in the smaller subunit (30S) of the prokaryotic ribosome, used in the mentioned method, is considered the best phylogenetic tool due to conserved fragments that are characteristic of certain taxonomic groups and enable the groups’ identification. The data from the literature showed that bacteria, with their host cells, form an interactive ecosystem of interdependencies and relationships. This influences both the metabolic (including, among others, metabolisms of phosphatidylcholine, lysophosphatidylcholine, and lysophosphatidylethanolamine [5]) and immune processes of the host [6,7]. Additional factors are gut permeability, bacteria translocation, and endotoxemia. All of the above-mentioned processes could contribute to CAD development as an important part of the pathogenetic process.

The gut microbiota of adults mostly consists of *Firmicutes* and *Bacteroidetes* that, together with *Actinobacteria* and *Proteobacteria,* account for nearly 99% of the intestinal microbiome [8]. Data from the literature indicate a significant change in the profile of the gut microbiome in various pathological conditions such as gastrointestinal diseases (inflammatory bowel diseases, irritable bowel syndrome) [9,10,11], autoimmune and inflammatory diseases (asthma, allergies, type 1 diabetes) [12,13], metabolic syndrome, type 2 diabetes, obesity [14,15], abnormal lipid profile [16], and hypertension [17].

Furthermore, a link between the intestinal microbiome and atherosclerosis of coronary arteries was also suggested [18,19,20]. A scientific report analyzing the microbial composition showed an increase in *Lactobacillales* and a decrease in *Bacteroidetes* (*Bacteroides* + *Prevotella*) in CAD patients compared with control and healthy groups [9,18]. The study of Zhu Q. et al. described an increase in *Escherichia-Shigella* and *Enterococcus* and a decrease in *Faecalibacterium*, *Subdoligranulum*, *Roseburia,* and *Eubacterium rectale* in the CAD group [20]. Furthermore, a study by Liu H. et al., showed that the abundance of several LPS-producing Gram-negative bacteria, such as *Veillonella*, *Haemophilus,* and *Klebsiella,* increased with CAD severity, while butyric-acid-producing bacteria such as *Lachnospiraceae* and *Ruminococcaceae* decreased with CAD development [21]. Another important study revealed that the taxa *Ruminococcus torques* in patients with stable CAD and type 2 diabetes mellitus have a predictive value in cardiac survival outcomes [22]. Moreover, it was shown that in altered CAD gut microbiome compositions [20], a higher *Firmicutes/Bacteroidetes* ratio plays an important role [18,23]. 

The study aims to assess the gut microbiome composition in the CAD patients in relation to a control group (without CAD, matched in terms of age and sex to the study group). Furthermore, we try to assess whether the marker of dysbiosis—*Firmicutes/Bacteroidetes* ratio—is related to biochemical and targeted metabolomics parameters.

## 2. Materials and Methods

### 2.1. Study Population

On the basis of the medical records, in the two-stage recruitment, 349 CAD patients aged 30–79 hospitalized 12–18 months prior to evaluation were identified. These CAD patients were hospitalized due to acute coronary syndrome (acute myocardial infarction with ST-segment elevation—STEMI, acute myocardial infarction with non-ST-segment elevation—NSTEMI, unstable angina/acute myocardial ischemia), or elective percutaneous coronary intervention (PCI) [24]. The inclusion and exclusion criteria used for recruitment are presented in Table 1. 

The 349 consecutive CAD patients hospitalized in three Departments of Cardiology, who fulfilled all inclusion criteria, were invited to participate in the study. Ninety-two patients did not respond to our invitation, so we do not know why they did not agree to participate; it is likely that some of them died. Two hundred fifty-seven patients arrived for an initial visit. A few patients, despite providing consent for gut microbiome analysis, did not provide a stool sample. Furthermore, a few patients provided incorrectly collected stool samples. 

The details of the participants’ medical history were collected from questionnaires at the time of the study entry. Starting in 2016, all patients underwent a detailed health assessment, with particular emphasis on the cardiovascular system. Furthermore, all of the participants were invited for a consultation visit, during which a physician discussed with them the obtained results. Patients were also asked to bring stool samples in the previously provided container (Stool Collection Tubes with Stool DNA stabilizer). From the stool sample, purified genomic DNA was isolated using the PSP-Spin Stool-DNA kit (Stratec), according to the manufacturer’s protocol. DNA was stored first at −20 °C, then −80 °C.

Finally, 169 CAD patients were included in the microbiome analysis. Further analyses were based on the V3–V4 region of the 16S rRNA gene (next generation sequencing) and bioinformatics analysis. Finally, the gut microbiome results were obtained for 169 CAD patients and 166 participants from the control group [25]. The control group consisted of 166 participants selected from inhabitants of the Bialystok. The goal of the selection algorithm was to maximize the *p*-value of the t-test for comparing age between patients and controls group and the *p*-value of Pearson’s chi-square test for comparing gender proportions. At the baseline visit, the control group received the same health assessment and stool sampling as the CAD group.

### 2.2. Quantitation of Metabolites Using an AbsoluteIDQ p180 Kit

Serum samples were stored at –80 °C until the day of analysis. On the day of analysis, samples were thawed on ice and then vortex-mixed for 1 min. Targeted analysis of serum samples was performed based on the procedure provided with the AbsoluteIDQ p180 kit (Biocrates Life Sciences AG, Innsbruck, Austria). Briefly, samples were prepared by adding 10 μL of the internal standards mixture supplied with the kit to each spot of a 96-well plate. Afterwards, 10 μL of calibration standard, QC samples, zero samples, and serum samples were added to the appropriate wells of the extraction plate. The plate was dried using the SpeedVac Concentrator (Savant SPD2010, Thermo Fisher Scientific, Waltham, MA, USA). After drying, derivatization with a mixture of ethanol, water, pyridine, and phenyl isothiocyanate was performed. Derivatization (25 min) was followed by drying the plate again. The analytes were extracted with 5 mM ammonium acetate in methanol. For LC-MS/MS analysis, sample extracts were diluted 1:1 with water, while for flow-injection MS/MS analysis (FIA-MS/MS), a 1:49 dilution with the Biocrates kit running solvent was performed.

Serum samples were quantified using ultrahigh performance liquid chromatography (1290 Infinity II, Agilent Technology, Santa Clara, CA, USA) coupled with a tandem mass spectrometer (6470 Triple Quad LC/MS, Agilent Technologies, Santa Clara, CA, USA) equipped with AJS-ESI ionization. The LC-MS/MS was operated in the multiple reaction monitoring (MRM) mode using positive (ESI+) ion mode. The specific MRM transitions for each analyte and internal standard were collected over the retention time window using the method provided with the kit. On the day of sample preparation, extracts from the LC part were injected for LC-MS measurements, while FIA-part analysis was performed the next day. 

Raw spectral data were loaded into Biocrates’ MetIDQ (Version 8.7.1, Biocrates, Life Science AG, Innsbruck, Austria) software, where the peaks of biogenic amines and amino acids (LC-Part) were integrated. After, the concentration calculation was performed using an external seven-point calibration curve based on isotope-labeled internal standards. The quantification of acylcarnitines, glycerophospholipids, sphingolipids, and hexose sum (FIA-part) was carried out by a one-point internal standard calibration. Quality of analyses was controlled by the injection of quality control samples on three concentration levels, of which the medium level of the quality control sample (QC2) was injected in three replicates. Triplicate of zero samples (PBS) was used to calculate the limits of detection (LOD). The median values of all zero samples were used to calculate background noise per metabolite signal, whereas three times this value was calculated as the LOD.

### 2.3. Statistical Analysis

Descriptive statistics was performed using the Statistica 13.1 software (StatSoft Polska, Cracow, Poland). The distribution of all variables was verified with the Kolmogorov–Smirnov test. Accordingly, based on distribution, parametric and non-parametric tests were used, and data were presented as medians and interquartile ranges or means and standard deviations. Statistical hypotheses were verified at the 0.05 significance level. 

The CLC Genomics Workbench program with the Microbial Genomics v21.0.1 module (QIAGEN, Aarhus A/S, http://www.clcbio.com accessed on 19 January 2021) was used to analyze the sequencing results of the gut microbiome. Identification of microorganisms to the genus level was conducted based on version 132 of the SILVA database, with the exclusion of the possibility to create operational taxonomic units (OTUs) de novo (“closed reference OTU picking” method). To infer functions from the taxonomic profiles, PICRUSt2 was used with default options [25]. The potential biomarkers were assessed with the use of the LEfSe method [26].

## 3. Results

### 3.1. Description of the Study Population

The final analysis included 169 CAD patients and 166 people from the control group, without CAD, matched in age and sex to the study group (Table 2). 

As expected, the CAD group was characterized by more frequent atherosclerotic plaques in the carotid arteries, higher total and android fat mass, and lower heart rate during the electrocardiogram due to pharmacotherapy (Table 2). Additionally, in this group, NTproBNP concentration was two times higher compared to the control (Table 3). In the CAD group, lower red blood cells count, hemoglobin, total cholesterol, low- and high-density lipoprotein cholesterol, and iron were shown (Table 3). Furthermore, in the CAD group, higher creatinine and urea, fasting glucose, and HbA1c, gamma-glutamyl transpeptidase were revealed (Table 3). Among echocardiographic parameters, only ejection fraction and left atrium diameter differ between study groups. The lower value of ejection fraction and larger left atrium were observed in the CAD population (Table 2).

### 3.2. Bioinformatic Biodiversity Analysis

**Figure 1:**
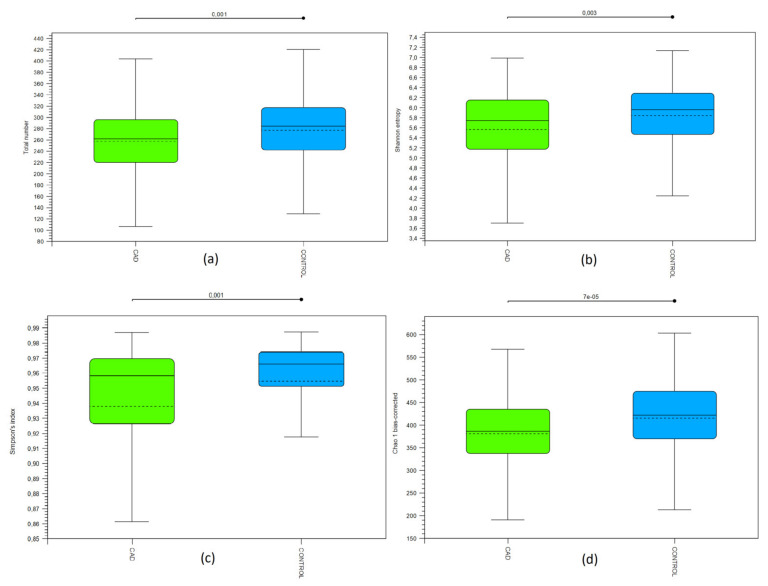
Alpha-biodiversity in coronary artery and control groups. CAD—coronary artery disease group. (**a**) The total amount of OTUs; OTUs—operational taxonomy units, (**b**) Shannon entropy, (**c**) Simpson Index, (**d**) Chao1bias-corrected estimator.

Bioinformatic analysis showed significant differences between the studied groups in terms of abundance (alpha-biodiversity). The CAD group was characterized by a lower amount of OTUs compared to the control (Figure 1a), lower Shannon entropy (Figure 1b), lower Simpson Index (Figure 1c), and Chao1bias-corrected estimator (Figure 1d).

Beta-biodiversity analysis showed significant differences between the studied groups (PERMANOVA based on the results of the Bray–Curtis metric; *p* < 0.001 using the Bonferroni correction).

### 3.3. Gut Microbiome Composition Analysis

In total, 12,837,581 good-quality paired reads were obtained from both populations and grouped into 4074 OTUs. After removing the rare OTUs (less than 10 readings obtained for all samples in total), 2893 OTUs were used for further analysis.

**Figure 2:**
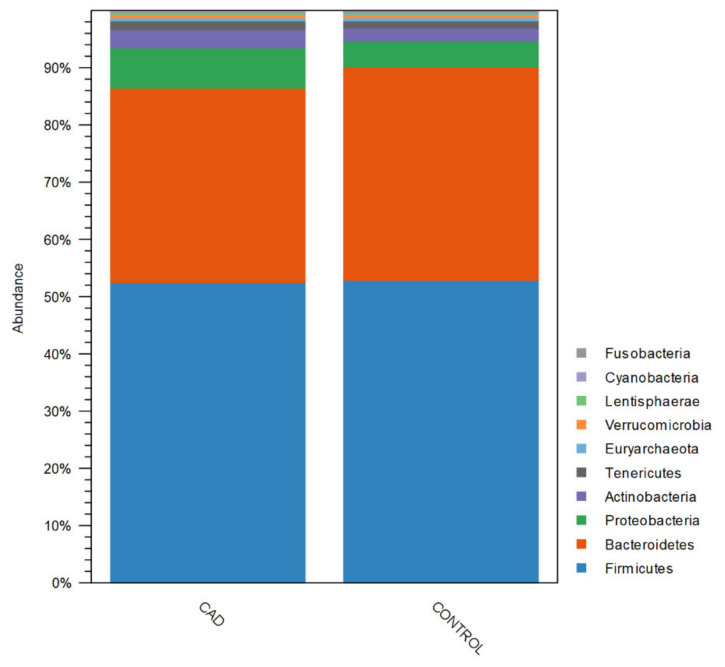
Composition of bacteria phyla in studied populations. CAD—coronary artery disease group.

Bioinformatics analysis showed that the most common bacterial phyla found in the gut microbiome of the studied populations were *Firmicutes*, *Bacteroidetes*, *Proteobacteria,* and *Actinobacteria* (Figure 2). 

**Figure 3:**
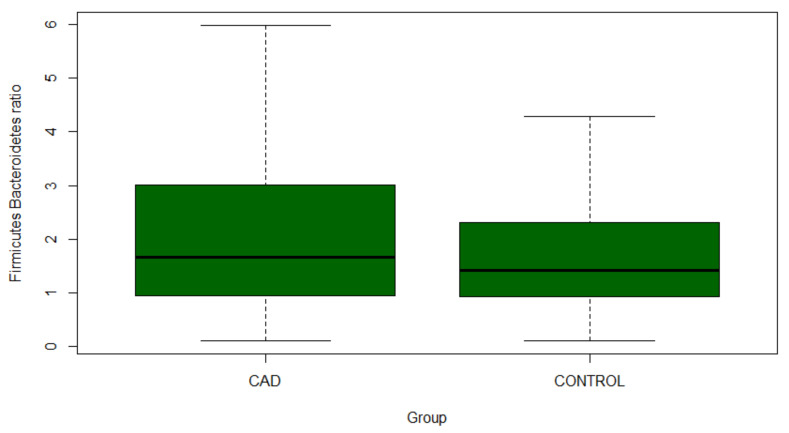
*Firmicutes/Bacteroidetes* ratio in CAD and control population. CAD—coronary artery disease.

In the CAD group, a higher *Firmicutes/Bacteroidetes* ratio compared to the control was demonstrated (Figure 3; median 1.67 IQR: 0.95–3.0 vs. 1.42 IQR: 0.94–2.3, *p* = 0.03).

At the phylum level, we did not find any difference in *Firmicutes* between the studied groups (Supplementary Table S1). The relative abundance of *Bacteroidetes* was significantly decreased, while *Proteobacteria* increased in the CAD group (Table 4). We observed a trend for the increased relative abundance of *Actinobacteria* in CAD patients, but the difference was not significant (Supplementary Table S1). We did not find differences in terms of other phyla (Supplementary Table S1).

The most common classes in both groups were *Clostridia* and *Bacteroidia* (Supplementary Figure S1, Supplementary Table S1). We have found a statistically significant increase in *Gammaproteobacteria* and *Bacilli* and a decrease in *Bacteroidia* in the CAD group (Table 4).

The most common orders in both groups were *Clostridiales* and *Bacteroidales* (Supplementary Figure S2, Supplementary Table S1). Moreover, a statistically significant increase in *Actinomycetales*, *Micrococcales*, *Lactobacillales*, *Enterobacteriales*, and decrease in *Bacteroidales* in the CAD group were found (Table 4).

The most common families were *Ruminococcsceae*, *Bacteroidaceae*, *Prevotellaceae,* and *Lachnospiraceae* (Supplementary Figure S3). Among all bacteria families, we found a significant increase in the relative abundance of *Actinomycetaceae*, *Micrococcaceae*, *Atopobiaceae, Lactobacillaceae*, *Streptococcaceae*, *Enterobacteriaceae,* and a decrease in *Barnesiellaceae* in the CAD group (Supplementary Table S2).

It is worth underlining that 31 among 43 genera that differ between study groups were *Clostridiales* from *Firmicutes* (Supplementary Table S2). 

Moreover, the most common microorganism in the entire (CAD and control) population, when considering the number of reads of the particular OTUs, was the genus *Faecalibacterium* of the *Firmicutes* (DQ808333.1.1386 SILVA, 359,139 reads). However, its abundance did not differ between study groups (*p* = 0.256).

Overall, we identified 1070 OTUs differentiating the studied groups, and 10 of them with the highest statistical significance are presented below (Table 5). Half of these microorganisms were in *Firmicutes* phylum. The most significant difference between studied populations was revealed for the *Clostridiales vadin BB60* group from *Firmicutes* (Table 5). Its lower value was presented in the CAD group (Table 5).

### 3.4. Prediction of the Functional Potential of the Intestinal Microbiome

**Figure 4:**
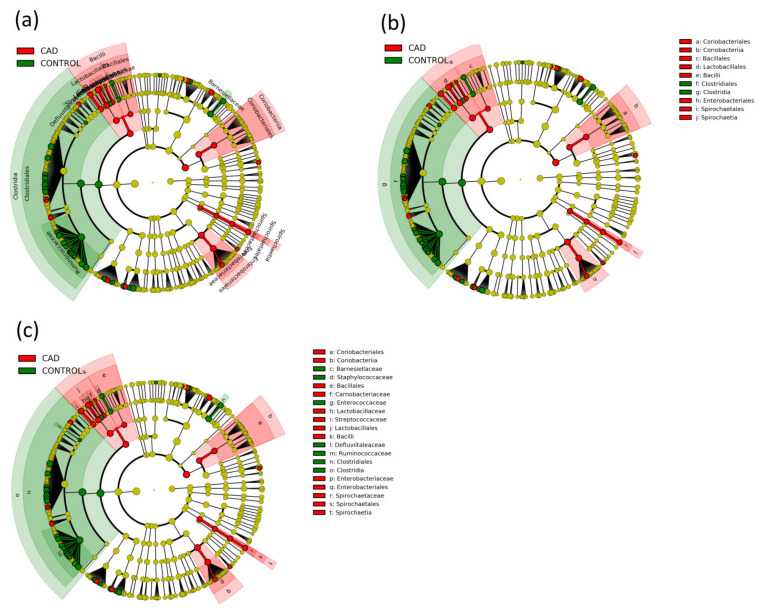
Cladogram showing various taxonomic changes in gut microbiota between the CAD and control groups: (**a**) general analysis; (**b**) in order level; (**c**) in order and class level.

Based on the obtained taxonomic profiles, with the use of PICRUSt2 (Phylogenetic Investigation of Communities by Reconstruction of Unobserved States) [26], the functional potential of the intestinal microbiome was predicted (Figure 4a–c).

**Figure 5:**
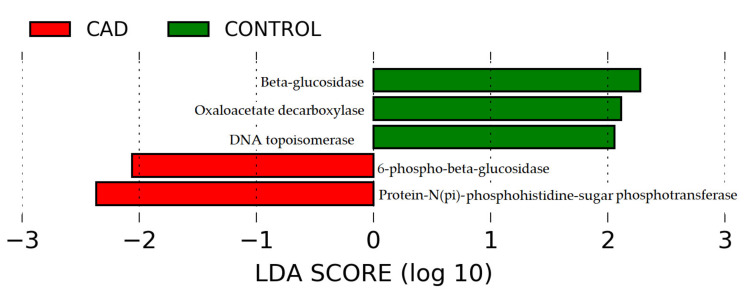
Potential changes in microbiome products in coronary artery disease and control groups at the protein level.

Then, using the LEfSe method [27], potential alterations in CAD compared to healthy controls were determined: 136 at the taxonomic level (Supplementary Figure S5), 55 in metabolic pathways (Supplementary Figure S6), and 5 at the protein level (Figure 5). Further, LEfSe showed that, among others, *Actinobacteria, Bacilli* and *Lactobacillales, Escherichia-Shigella, Streptococcus, Enterobacteriales, Coriobacteria,* and *Spirochateacea* were more abundant in the CAD group. In contrast, *Clostridia, Lachnospiraceae, Selenomonales, Ruminococcaceace*, and *Bacteroides* were more abundant in the control group (Supplementary Figure S5).

Based on this indirect analysis, the microbiome of the control group is expected to participate more in vitamin (adenosylcobalamin, thiamin, riboflavin); amino acid (glutamine, arginine, asparagine, aspartate, serine, glycine); and saccharide (mannan, glycogen, gluconeogenesis, starch, fructuronate, galacturonate) pathways, whereas the CAD gut microbiome participates more in vitamin (menaquinol); hydroxy acid (mevalonate); amino acid (alanine, methionine, aspartate, threonine); polysaccharide (hexitol); and nucleoside (inosine, adenosine, guanosine) metabolism (Supplementary Figure S6). 

In both groups, Clostridia and Bacterioidia potentially play a role in DNA topoisomerase, oxaloacetate decarboxylase, and 6-beta-glucosidase production (Supplementary Figures S7–S9), while Clostridia and Gammaproteobacteria might play a role in 6-phospho-beta-glucosidase and protein-N(pi)-phosphohistidine-sugar phosphotransferase production (Supplementary Figures S10 and S11).

### 3.5. Targeted Metabolomics and Biochemical Parameters

The median value of the *Firmicutes/Bacteroidetes* ratio in the whole study population (CAD and control groups) was defined at the level of 1.54. A higher *Firmicutes/Bacteroidetes* ratio after adjustment for age, sex, CAD status, LDL cholesterol concentration, and statins treatment was characterized by lower phosphatidylcholine with diacyl residue and sphingomyelin with acyl residue, whereas a higher concentration of hemoglobin, hematocrit, and two among three phosphatidylcholines with acyl-alkyl residue (Table 6). Furthermore, this group was characterized by lower total and LDL cholesterol after adjustment for age, sex, CAD status, and statins treatment (Table 6). There were no other statistically significant differences between the groups with a *Firmicutes/Bacteroidetes* ratio below and above the median in the context of other targeted metabolomes (Supplementary Table S3).

## 4. Discussion

The most important finding of the article is that the gut microbiome differs between CAD and control groups. 

As expected, our results confirmed some of the findings from the literature in the aspect of microbiome composition. In our population, independent of the history of CAD, the most common bacterial phyla in the gut microbiome were *Firmicutes*, *Bacteroidetes*, *Proteobacteria,* and *Actinobacteria* [10,28]. At a phylum level, an increased relative abundance of *Proteobacteria* and *Actinobacteria* and a decreased abundance of *Firmicutes* and *Bacteroidetes* were revealed [20]. We confirmed the above-mentioned shifts of the microbiome with the exception of *Firmicutes* and only a trend for *Actinobacteria.*

Due to the numerous bacteria within each phylum, and their impact related to the health and individual features of the host, it is difficult to clearly define whether they are positive or negative. However, they seem to be related to different cardiovascular risk factors and diseases. *Bacteroidetes* are generally considered favorable, while *Firmicutes’* role is ambiguous [29]. 

Most bacteria of the *Firmicutes* phylum have a Gram-positive cell wall structure. Its high abundance was connected to the indicators of unhealthy lifestyles, such as a high-fat diet [30], abnormal energy balance [31,32], and obesity [33]. On the other hand, some bacteria from this phylum are short-chain-fatty-acid (SCFA) producers, such as acetate, butyrate-producing species, that seems to have a beneficial impact on health by stimulating the host’s immune system, modulating metabolic health, and improving metrics of cardiovascular risk factors such as blood pressure, metabolism, and the integrity of the intestinal barrier [34]. Among butyrate producers, the most abundant species (in terms of reads, *DQ808333.1.1386*) in our population is *Faecalibacterium* from *Ruminococcaceae*. Although we did not reveal a decrease in *Ruminococcaceae* in CAD, it was revealed in both the article of Zhu Q. et al. [20] and Liu H. et al. [21]. According to the literature, *Faecalibacterium prausnitzii* was found to be the most abundant bacterium in the human intestinal microbiota of healthy adults, representing more than 5% of the total bacterial population [35]. Although this particular species did not differ between study groups, a higher relative abundance of the *Faecalibacterium* genus was found in the control group. 

Another SCFAs-producing bacteria—*Clostridiales vadin BB60,* which was the most significantly different between study groups, was characterized by a higher abundance in the control population. Similarly, in the other work, this bacterium decreases with CAD development [20,21]. It has been shown that the *Clostridiales vadin BB60 group* [36,37] is inversely correlated with obesity, dyslipidemia, and insulin resistance in the mice model [38,39] and with BMI, weight, and waist in women [40]. Its reduction was also associated with high TMAO levels and thrombotic risk [41,42,43]. On the other hand, its increased abundance was shown in women with hyperglycemia in pregnancy [40]. 

Furthermore, in this study, similarly to other authors, we found an increase in the abundance of other protective taxa from *Firmicutes-Lactobacillales* from *Bacilli* in the CAD group [9,18,20]. These bacteria are characterized by lactic acid production as the main product of glucose and by growth inhibition substances such as bacteriocins, hydrogen peroxide, and diacyls [44]. These substances prevent the proliferation of food spoilage bacteria and pathogens [44]. 

Additionally, when we consider the genus level of *Firmicutes,* our results are consistent with the work of Zhu Q. et al., in which stool samples were collected from 70 patients with coronary artery disease and 98 healthy controls [20]. As in the mentioned study, we showed that *Faecalibacterium, Roseburia* dominated the control group compared to the CAD group, while *Escherichia-Shigella* were enriched bacteria in CAD patients [20]. 

The above-mentioned potentially pathological taxa from *Proteobacteria* such as *Escherichia-Shigella* were proven to increase in CAD patients [20]. It was demonstrated that the abundance of several Gram-negative bacteria, producing LPS, such as *Haemophilus* and *Klebsiella,* increased with CAD severity [21]. We did not observe any differences in the relative abundance of these genera; however, we confirmed an increase in *Gammaproteobacteria* and *Enterobacteriales*, *Enterobacteriaceae* in the CAD group. Moreover, we suspected a potential *Gammaproteobacteria* impact of bacterial 6-phospho-beta-glucosidase and protein-N(pi)-phosphohistidine-sugar phosphotransferase production.

Although the data concerning particular microbiota that alter in the CAD group mostly concern *Firmicutes*, there is some information on *Bacteroidetes* as well. The phylum *Bacteroidetes* is composed of Gram-negative bacteria, whose abundance increases with fiber consumption [45], a high-carbohydrate diet [46], and decreases with atherosclerosis progression [47]. This phylum includes bacteria producing SCFA such as acetate and propionate [34]. The scientific reports analyzing the microbial composition showed a decrease in *Bacteroidetes* (*Bacteroides* + *Prevotella*) in CAD patients compared to control [9,18,23]. Based on these findings, a preclinical study identified that oral gavage with live *B. vulgatus* and *B. dorei* attenuated the development of atherosclerosis in atherosclerosis-prone mice [48]. In our study, we confirm a significant decrease in *Bacteroidetes, Bacteroidia,* and *Bacteroidales* in CAD. However, we did not observe any differences in *Prevotella,* except for the genus *Paraprevotella.*

*Prevotella* was associated with a diet rich in non-digestible carbohydrates, such as fiber [49], and, despite its positive value, it seems to also have a negative influence on health. It played an important role in dysbiosis in pre- and hypertension patients [50]. This genus was also enriched among high lifetime cardiovascular disease risk profile Bogalusa Heart Study participants [51], and *Prevotella copri* was proven to be associated with cardiac valve calcification [52]. Moreover, in predicted functional analysis, *Bacteroidia* seems to play a role in DNA topoisomerase, oxaloacetate decarboxylase, and 6-beta-glucosidase production.

Although we only showed a trend in the increase in *Actinobacteria* phylum in the CAD group, we confirmed a statistically significant increase in the *Actinomycetales* and *Micrococcaceae* order. 

Although findings regarding particular microorganisms profiles in CAD differ between studies, the most common result is an increase in *Firmicutes/Bacteroidetes* ratio and reduction in microbial diversity and richness [18,23,53,54]. This ratio is considered an indicator for gut dysbiosis. It was proven that the *Firmicutes/Bacteroidetes* ratio was significantly associated with many traditional cardiovascular risk factors such as diet [18,55], sex [56,57,58], age [59], and BMI [60]. Similarly, the *Firmicutes/Bacteroidetes* ratio value (not exceeding 2) was previously reported by Emoto T. et al. [18].

To verify the importance of the *Firmicutes/Bacteroidetes* ratio, we performed statistical analyses, including its value and targeted metabolome profile. We reveal that when we exclude the influence of age, sex, CAD status, LDL cholesterol concentration, and statins treatment, the *Firmicutes/Bacteroidetes* ratio is mostly related to phosphatidylcholine and sphingomyelin with its lower value in several phosphatidylcholines in a higher *Firmicutes/Bacteroidetes* ratio. To our knowledge, this is the first study that tries to reveal whether the *Firmicutes/Bacteroidetes* ratio, independent of main disease and other cofactors, might cause changes in metabolome. A previous study underlined that several phosphatidylcholines were lower in type 2 diabetic subjects with CAD [61] and in patients with silent myocardial ischemia as a consequence of coronary heart disease [62]. The authors suggest that lower serum phosphatidylcholine levels resulted from increased uptake, which is necessary for reconstructing damaged cardiomyocytes membrane due to the pathological process [61] and is a result of insufficient supply of ATP and CTP [62].

Interestingly, in our population, we identified more high-quality OTUs than in the literature (2893 vs. 1101 in the work of Zhu Q. et al. [20] and vs. 1308 OTUs in the article by Zheng Y.Y. et al. [28]). Due to the lack of influence of the number of OTUs by the NGS method, we suspected we only identified and included good-quality samples in the final analyses. According to the data concerning alpha-biodiversity presented in previous studies [20,55], in this study, in the CAD group, we showed a decreased number of OTUs as well as differences in alpha and beta-diversity.

Despite some differences in particular species and genera that we prove relevant in our study, generally, we found a similar gut microbiome pattern in the study that was presented in most of the previous articles, with the exception of the article by Zheng Y. et al. [28]. However, this article was based on the Chinese population with stable CAD, which might be the reason for different findings.

Interesting findings from our study are results from predicted functional microbiome analysis. In this study, we tried to not only identify particular potential alterations in CAD patients but also to provide some hypothetical relation between biomarker microorganisms, pathways, and their products. In the study, using the LEfSe method, we identified bacteria that differ between study groups. Our findings in terms of taxa identification are in line with the findings of Zhu Q. et al. and Toya T. et al. [20,54], and again are contrary to previously mentioned findings provided by Zheng Y.Y. et al. [28].

Importantly, in our article, all three potential product markers for the control group were connected to *Clostridia* and *Bacterioidia,* whereas those related to the CAD group were *Clostridia* and *Gammaproteobacteria.*

A beta-glucosidase (EC 3.2.1.21, cellobiase) was one of three potential control group markers. This enzyme degrades cellobiose units and oligodextrin to glucose [63]. Oxaloacetate decarboxylase (EC 4.1.1.3) is involved in the conversion of oxaloacetate into pyruvate [64]. The final product of glycolysis, pyruvate, is converted to acetyl-CoA and CO_2_ by the action of the pyruvate dehydrogenase complex. The product of the complex, acetyl-CoA, feeds into the TCA cycle I, a catabolic pathway that generates energy and reduces power as well as precursors for biosynthesis. In every turn, the TCA cycle converts one molecule of acetyl-CoA into two CO_2_ molecules, reduces a total of four molecules of either NAD+, NADP+, or quinone to NADH, NADPH, and quinol, respectively, and phosphorylates one molecule of GDP to GTP [65]. DNA topoisomerase (EC 5.99.1.2) relaxes supercoiled DNA without a divalent cation or ATP and might represent a potential mechanism for the action of the antibiotics [66].

The 6-phospho-β-glucosidase BglA-2 (EC 3.2.1.86, CAD group biomarker), identified from *Streptococcus* (CAD group biomarker) *pneumoniae TIGR4* and *pyogenes* [67], is a member of glycoside hydrolase family 1 that catalyzes the hydrolysis of β-1,4-linked cellobiose 6-phosphate (cellobiose-6′P) to yield glucose and glucose 6-phosphate. Both reaction products are further metabolized by the energy-generating glycolysis pathway [68]. This taxonomic biomarker participates in the pathway of starch and gluconeogenesis [68,69].

Protein-N(pi)-phosphohistidine-sugar phosphotransferase (EC 2.7.1.69, CAD group biomarker) is an enzyme that catalyzes the chemical reaction where protein histidine and sugar-phosphate are produced from Npi-phospho-L-histidine and sugar. It is produced by various microorganisms [70], and some of them, such as *E. coli*, from *Gammaproteobacteria,* were identified in the CAD group. This enzyme participates in metabolic pathways, among which gluconeogenesis, fructose, mannose, and galactose metabolism were found in the control group [71].

Moreover, in our study, the CAD group is connected to many pathways (superpathway of menaquinol-8 biosynthesis I, of menaquinol-7, superpathway of demethylmenaquinol-8 biosynthesis, of demethylmenaquinol-9 biosynthesis, of menaquinol-11 biosynthesis, of menaquinol-12 biosynthesis, of menaquinol-13 biosynthesis, of demethylmenaquinol-6 biosynthesis II) of nitric oxide reduction to nitrous oxide in which menaquinone (vitamin K) and H_2_O are produced. Interestingly, in the literature, menaquinone was suggested to reduce the incidence of coronary heart disease [72,73].

Microorganisms from the CAD group also take part in the superpathway of geranylgeranyl diphosphate biosynthesis I (via mevalonate) and mevalonate pathway I. Mevalonate acid plays a role in the biosynthesis of sterols and isoprenoids. The mevalonate pathway produces isopentenyl diphosphate, a building block for polyisoprenoid synthesis, and is a crucial pathway for the growth of the human bacterial pathogen *Enterococcus faecalis* [74].

## 5. Limitation of the Study

The study’s main limitation was the limited number of patients and stool samples that were of sufficient quality for further microbiome analysis. The most common reason for the inadequate DNA quality that leads to an insufficient number of high-quality nucleotide sequences was the patient’s incorrect collection of a stool sample. Furthermore, some samples had to be resequenced due to technical problems during sequencing.

Another limitation is gut microbiome susceptibility to many environmental factors, such as diet and medication. On the other hand, this is also the reason for performing replication studies such as ours in different populations and environments. To our knowledge, this is one of the largest studies on the CAD gut microbiome concerning a population exceeding 330 patients (169 CAD patients and 166 controls). Our study confirmed previously reported gut microbiome changes in a pattern of environmental and genetic factors of Eastern and Central Europe. Other studies concerning similar topics were based on 152 CAD patients and 105 healthy controls [28] and on 161 CAD patients and 40 healthy controls [21]. Moreover, the latter study revealed that the gut microbiota composition changed significantly with CAD severity [21].

In this study, we were able to provide sex- and age-matched controls. Unfortunately, we were not able to select similar groups in terms of all clinical data, such as metabolic syndrome, diabetes, etc., that might influence the gut microbiome. Nevertheless, we excluded other diseases that might affect the microbiome, such as active cancer or history of intestinal acute or chronic disease. Furthermore, we want to underline that a lack of symptoms does not exclude the presence of atherosclerotic plaques. Hence, in such a control group (matched to the CAD group in terms of many cardiovascular risk factors), there could be a high proportion of silent CAD patients. This issue could be addressed only in a large, prospective study. 

Furthermore, in this study, we performed hypothetical predicted functional analyses based on the gut microbiota’s potential to produce metabolites. 

We were able to perform targeted metabolome experiments using Biocrates, not untargeted metabolomics fingerprinting. We did not perform Principal Coordinates Analysis and did not test described pathways in experimental mouse models. These might be interesting in further follow-ups to this project studies.

## 6. Conclusions

There are marked differences in the gut microbiome between the CAD and control groups that may translate into different metabolisms and hence may affect the development of atherosclerosis. Furthermore, the *Firmicutes/Bacteroidetes* ratio, independent of CAD status and several confounders, is related to altered targeted metabolome profiles. Thus, a gut microbiome composition assessment might become a useful tool in cardiovascular risk stratification in the future due to its relation to biochemical and metabolome parameters. In order to precisely determine gut microbiome impact in CAD pathogenic process, a functional analysis with advanced, novel “omics” methods should be conducted in a large, prospective study.

## Figures and Tables

**Table 1:** The inclusion and exclusion criteria.

	CAD Group	Control Group
Inclusion criteria	- Signed informed consent - Aged 30–79 years	
	- Hospitalization 12–18 months prior to the evaluation for: - Elective percutaneous coronary intervention - Acute coronary syndromes - ST-elevation myocardial infarction (STEMI) Non-ST elevation myocardial infarction ST (NSTEMI) Unstable angina (UA)/acute myocardial ischemia	- Randomly selected from the Bialystok population - SEX and age matched to CAD group - Without CAD - Asymptomatic patients in terms of angina - Without history of coronary angiography, that revealed atherosclerotic plaques
Exclusion criteria	- History of intestinal acute or chronic disease - Active cancer - Aged below 30 and over 80 - Lack of informed consent	
		- Angiographic confirmation of CAD - History of acute coronary syndrome - Typical angina

CAD—coronary artery disease.

**Table 2:** Characteristics of study groups.

	CAD Group (*n* = 169)	Control Group (*n* = 166)	*p* Value
Age, years	64.1 ± 7.7	62.4 ± 10.5	0.08
Sex, male, *n* (%)	124 (73%)	108 (65%)	0.1
BMI, kg/m^2^	30.52 ± 5.3	28.46 ± 4.73	0.001
WHR	0.96 ± 0.08	0.92 ± 0.09	0.001
Arterial hypertension, *n* (%)	138 (83%)	82 (56%)	0.001
Diabetes, *n* (%)	50 (30%)	16 (15%)	0.003
Current tobacco smoker, *n* (%)	28 (22%)	26 (27%)	0.435
History of tobacco smoking, *n* (%)	125 (74%)	98 (60%)	0.005
Lower limb atherosclerosis or intermittent claudication, *n* (%)	12 (80%)	3 (3%)	0.001
Hypercholesterolemia, *n* (%)	137 (82%)	69 (50%)	0.001
History of kidney disease, *n* (%)	14 (9%)	14 (11%)	0.435
Family history of the acute coronary syndrome, *n* (%)	44 (30%)	10 (7%)	0.001
Family history of stroke, *n* (%)	33 (22%)	17 (13%)	0.037
**Pharmacotherapy**			
Use of ACE-I/ARB, *n* (%)	147 (88%)	56 (44%)	<0.001
Use of beta-blockers, *n* (%)	53 (31%)	23 (18%)	0.012
Use of statins, *n* (%)	150 (90%)	38 (30%)	<0.001
Use of diuretics, *n* (%)	84 (50%)	18 (14%)	<0.001
Use of antiplatelet and anticoagulant treatment, *n* (%)	164 (98%)	30 (24%)	<0.001
**Assessments**			
Atherosclerotic plaques in the carotid arteries, *n* (%)	163 (96%)	123 (74%)	0.001
Heart rate in electrocardiogram, beats/minute	62 IQR: 58–68.5	64.5 IQR: 58–72	0.044
**Bone Densitometry**			
Total fat mass, g	29667.93 IQR: 23652.98–35995.84	28604.12 IQR: 20777.52–33493.96	0.047
Android fat mass, g	3220.41 IQR: 2510.92–4005.02	2849.09 IQR: 1991.26–3752.75	0.002
**Echocardiography**			
Left ventricle ejection fraction, %	52.3 IQR: 47.6–57.9	57 IQR: 53.8–60.4	<0.001
Left atrium diameter, mm	39.8 IQR: 36–43.9	37.3 IQR: 33.2–39.7	<0.001

Mean ± standard deviation, median IQR—interquartile range, BMI—body mass index, and WHR—waist–hip ratio.

**Table 3:** Biochemical tests.

Biochemical Test			
NTproBNP, pg/mL	154.5 IQR: 78.1–329	65.2 IQR: 32.6–110.1	0.001
WBC, tys/μL	6.4 IQR: 5.2–7.5	5.9 IQR: 5.1–6.9	0.119
RBC, mln/mm3	4.7 IQR: 4.4–5	4.9 IQR: 4.5–5.08	0.012
Hb, g/dL	14.1 IQR: 13.1–14.6	14.7 IQR: 13.7–15.4	<0.001
PLT, tys/μL	217 IQR: 185–256.5	214 IQR: 185–249	0.985
Creatinine, μmol/L	85.4 IQR: 74.4–97.2	75.8 IQR: 64.9–83.7	<0.001
Urea, mg/dL	34.4 IQR: 30–41	32.6 IQR: 28.4–38.7	0.011
Fasting glucose, mg/dL	105 IQR: 97–119.5	102 IQR: 96–109	0.027
120 min glucose, mg/dL	130.5 IQR: 102–161	134 IQR: 106–174	0.398
HbA1c, %	5.9 IQR: 5.6–6.2	5.7 IQR: 5.3–5.9	<0.001
Total cholesterol, mg/dL	154 IQR: 129.5–179	195 IQR: 166–227	<0.001
Triglycerides, mg/dL	103 IQR: 73–157	113 IQR: 82–149	0.394
Low-density lipoprotein cholesterol, mg/dL	85.8 IQR: 69.4–108.8	132.6 IQR: 99.9–160.8	<0.001
High-density lipoprotein cholesterol, mg/dL	48 IQR: 41.5–61.5	58 IQR: 47.4–68.3	<0.001
hs-CRP, mg/L	1.1 IQR: 0.5–2.1	0.9 IQR: 0.5–1.7	0.138
ALT, IU/L	23 IQR: 18.3–32.3	21.1 IQR: 16.9–29.6	0.05
AST, IU/L	23 IQR: 18.8–27.9	22.3 IQR: 19.4–26.9	0.167
GGT, IU/L	23.9 IQR: 16.3–39.1	20.2 IQR: 13.6–34.7	0.033
Iron, μg/mL	99.7 IQR: 82–119.7	113.8 IQR: 88.7–133.8	0.006

Mean ± standard deviation, median IQR—interquartile range. WBC—white blood cell, RBC—red blood cell, Hb—hemoglobin, PLT—platelets, CRP—C-reactive protein, ALT—alanine aminotransferase, AST—aspartate aminotransferase, and GGT—gamma-glutamyltranspeptidase.

**Table 4:** Statistically significant differences in relative abundance of bacterial phyla, classes, and orders in studied populations.

Taxonomic Unit	Relative Abundance in CAD Group	Relative Abundance in Control Group	*p*-Value (Adjusted for Sex and Age)
**Phyla**			
*Proteobacteria*	7.4695 (5.1935–9.7455)	4.6714 (3.4979–5.8449)	0.032
*Bacteroidetes*	33.5334 (30.8517–36.2151)	37.1957 (35.1636–39.2278)	0.032
**Classes**			
*Bacteroidetes, Bacteroidia*	33.5334 (30.8517–36.2151)	37.1957 (35.1636–39.2278)	0.032
*Firmicutes, Bacilli*	4.1117 (2.9382–5.2852)	1.2206 (0.7898–1.6514)	<0.001
*Proteobacteria, Gammaproteobacteria*	6.0816 (3.8101–8.3531)	3.4073 (2.2397–4.5749)	0.04
**Order**			
*Actinobacteria, Actinobacteria, Actinomycetales*	0.0281 (0.0164–0.0398)	0.0135 (0.0095–0.0175)	0.021
*Actinobacteria, Actinobacteria, Micrococcales*	0.0152 (0.0108–0.0196)	0.0083 (0.0043–0.0123)	0.021
*Bacteroidetes, Bacteroidia, Bacteroidales*	33.4714 (30.7887–36.1541)	37.1229 (35.0883–39.1575)	0.033
*Firmicutes, Bacilli, Lactobacillales*	3.891 (2.7367–5.0453)	1.1638 (0.745–1.5826)	<0.001
*Proteobacteria, Gammaproteobacteria, Enterobacteriales*	5.4589 (3.1754–7.7424)	2.5555 (1.4063–3.7047)	0.026

CAD—coronary artery disease group, CI—confidence interval. Mean values together with the 95% confidence intervals of the means are presented.

**Table 5:** OTUs with the highest statistical significance that differ between study groups.

Phylum	Order	Family	Genus	CAD Group (*n* = 169)	Control Group (*n* = 166)	Adjusted *p*-Value (FDR) CAD vs. Control
*Firmicutes*	*Clostridiales*	*Clostridiales* *vadinBB60 group*	*Uncultured organism*	0.3797 (0.1217–0.6377)	0.4296 (0.2595–0.5997)	<0.001
*Fusobacteria*	*Fusobacteriales*	*Fusobacteriaceae*	*Fusobacterium*	0.159 (0.0099–0.3081)	0.2248 (−0.0595–0.5091)	<0.001
*Firmicutes*	*Lactobacillales*	*Lactobacillaceae*	*Lactobacillus sp., Gut metagenome*	0.1493 (−0.1451–0.4437)	0.0002 (0–0.0004)	<0.001
*Firmicutes*	*Clostridiales*	*Christensenellaceae*	*Christensenellaceae* *R-7 group*	1.3802 (1.0337–1.7267)	1.1589 (0.8781–1.4397)	<0.001
*Firmicutes*	*Lactobacillales*	*Lactobacillaceae*	*Lactobacillus sp, Ambiguous taxa*	0.7075 (0.2925–1.1225)	0.0626 (0.0121–0.1131)	<0.001
*Spirochaetes*	*Spirochaetales*	*Spirochaetaceae*	*Treponema 2*	0.0699 (−0.0293–0.1691)	0 (0–0)	<0.001
*Bacteroidetes*	*Bacteroidales*	*Prevotellaceae*	*Prevotellaceae UCG-003*	0.0434 (−0.0297–0.1165)	0.0002 (−0.0002–0.0006)	<0.001
*Firmicutes*	*Clostridiales*	*Ruminococcaceae*	*Ruminococcaceae UCG-014*	4.6675 (3.6518–5.6832)	3.5864 (2.8496–4.3232)	<0.001
*Bacteroidetes*	*Bacteroidales*	*Muribaculaceae*	*Uncultured bacterium*	0.108 (0.027–0.189)	0.1365 (0.0385–0.2345)	<0.001
*Proteobacteria*	*Enterobacteriales*	*Enterobacteriaceae*	*Escherichia-Shigella*	4.5528 (2.398–6.7076)	1.9492 (0.9105–2.9879)	<0.001

CAD—coronary artery disease group, OTUs—operational taxonomic units. Mean values together with the 95% confidence intervals of the means are presented.

**Table 6:** The influence of *Firmicutes/Bacteroidetes* ratio on targeted metabolomics (Biocrates) and biochemical test adjusted for age, sex, CAD status, statins treatment (and) LDL cholesterol concentration.

Metabolite or Biochemical Test	Group with F/B Ratio ≤ 1.54 (*n* = 167)	Group with F/B Ratio > 1.54 (*n* = 167)	*p* Value
Phosphatidylcholine with diacyl residue sum C36:4, μmol/L	145,308.5375 (95% CI 136,165.244–154,451.831)	131,525.3521 (95% CI 122,485.2512–140,565.4529)	0.037 *
Phosphatidylcholine with diacyl residue sum C38:5, μmol/L	46,853.2875 (95% CI 43,791.1361–49,915.4389)	41,838.3521 (95% CI 39,090.319–44,586.3852)	0.019 *
Phosphatidylcholine with diacyl residue sum C38:6, μmol/L	68,122.05 (95% CI 62,784.975–73,459.125)	58,899.7887 (95% CI 54,099.1744–63,700.403)	0.013 *
Phosphatidylcholine with diacyl residue sum C40:6, μmol/L	26,837.9747 (95% CI 24,778.9683–28,896.9811)	23,893.6056 (95% CI 21,873.7272–25,913.484)	0.047 *
Phosphatidylcholine with acyl-alkyl residue sum C30:0, μmol/L	165.6974 (95% CI 153.7441–177.6507)	209.942 (95% CI 178.6529–241.2311)	0.008 *
Phosphatidylcholine with acyl-alkyl residue sum C34:0, μmol/L	839.25 (95% CI 782.9649–895.5351)	958.2254 (95% CI 852.6987–1063.7521)	0.045 *
Phosphatidylcholine with acyl-alkyl residue sum C38:0, μmol/L	1663.75 (95% CI 1520.5806–1806.9194)	1465.4225 (95% CI 1353.087–1577.758)	0.034 *
Sphingomyelin with acyl residue sum C24:1, μmol/L	69,876.9375 (95% CI 65,633.3358–74,120.5392)	63,321.1268 (95% CI 59,226.511–67,415.7426)	0.028 *
Hematocrit, %	41.3928 (95% CI 40.8434–41.9422)	41.8814 (95% CI 41.3236–42.4392)	0.047 *
Hemoglobin, g/dL	14.1371 (95% CI 13.9429–14.3313)	14.3413 (95% CI 14.1502–14.5324)	0.023 *
Total cholesterol, mg/dL	188.9341 (95% CI 181.2163–196.6519)	171.2455 (95% CI 164.3927–178.0983)	0.006 **
LDL cholesterol, mg/dL	118.8796 (95% CI 112.3616–125.3976)	104.9257 (95% CI 98.4497–111.4017)	0.026 **

CI—confidence interval. Mean values together with the 95% confidence intervals of the means are presented. (* Adjusted for Age, Sex, CAD Status, LDL Cholesterol Concentration, Statins Treatment; ** Adjusted for Age, Sex, CAD Status, Statins Treatment).

## Data Availability

The data set we generated during and/or analyzed during the current study are not publicly available due to confidentiality issues but are available from the corresponding author on request.

## Supplementary Materials

The following are available online at https://www.mdpi.com/article/10.3390/jcm10215074/s1. Figure S1: Composition of bacteria class in studied populations. CAD—coronary artery disease group. Figure S2: Composition of bacteria order in studied populations. CAD—coronary artery disease group. Figure S3: Composition of bacteria family in studied populations. CAD—coronary artery disease group. Figure S4: Composition of bacteria genus in studied populations. CAD—coronary artery disease group. Figure S5: Potential biomarkers for coronary artery disease and control group at the taxonomic level. LEfSe identified the major bacteria at all taxonomic levels at the threshold of absolute LDA score 2. Figure S6: Potential biomarkers for coronary artery disease and control group at pathways level. LEfSe identified the major bacteria at all taxonomic levels at the threshold of absolute LDA score 2. Figure S7: Potential DNA topoisomerase producers in coronary artery disease patients (a) and control group (b). Figure S8: Potential oxaloacetate decarboxylase producers in coronary artery disease patients (a) and control group (b). Figure S9: Potential beta-glucosidase producers in coronary artery disease patients (a) and control group (b). Figure S10: Potential 6-phospho-beta-glucosidase producers in coronary artery disease patients (a) and control group (b). Figure S11: Potential protein-N(pi)-phosphohistidine-sugar phosphotransferase producers in coronary artery disease patients (a) and control group (b). Table S1: The differences in relative abundance of bacterial phyla, classes, and orders in studied populations. Table S2: Statistically significant differences in the relative abundance of bacterial families and genera in studied populations. Table S3: The influence of *Firmicutes/Bacteroidetes* ratio on targeted metabolomics (Biocrates) and biochemical test adjusted for age, sex, CAD status, statins treatment (and) LDL cholesterol concentration.
