# Supplementary material for: Gut Microbiome in Chronic Coronary Syndrome Patients
Source: J Clin Med. 2021 Oct 29;10(21):5074. doi: 10.3390/jcm10215074 (PMC8584954; doi:10.3390/jcm10215074)
Supplement: Supplementary file 1 [file jcm-10-05074-s001.zip › jcm-1385699 Supplementary materials .pdf]

Journal of
Clinical Medicine

MDPI

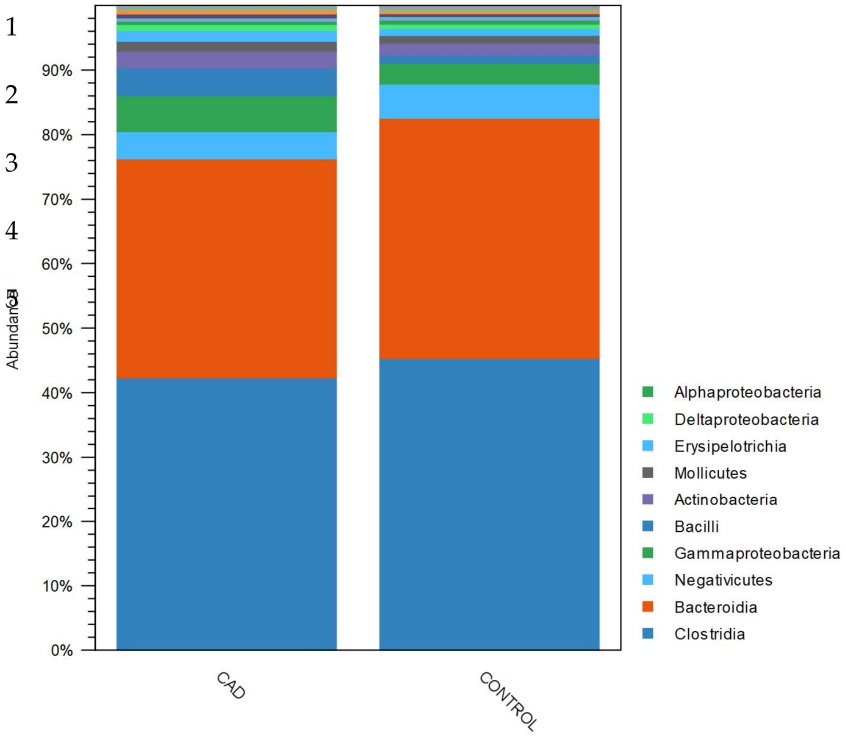

Supplementary Figure S1. Composition of bacteria class in studied populations. CAD – coronary artery disease group.

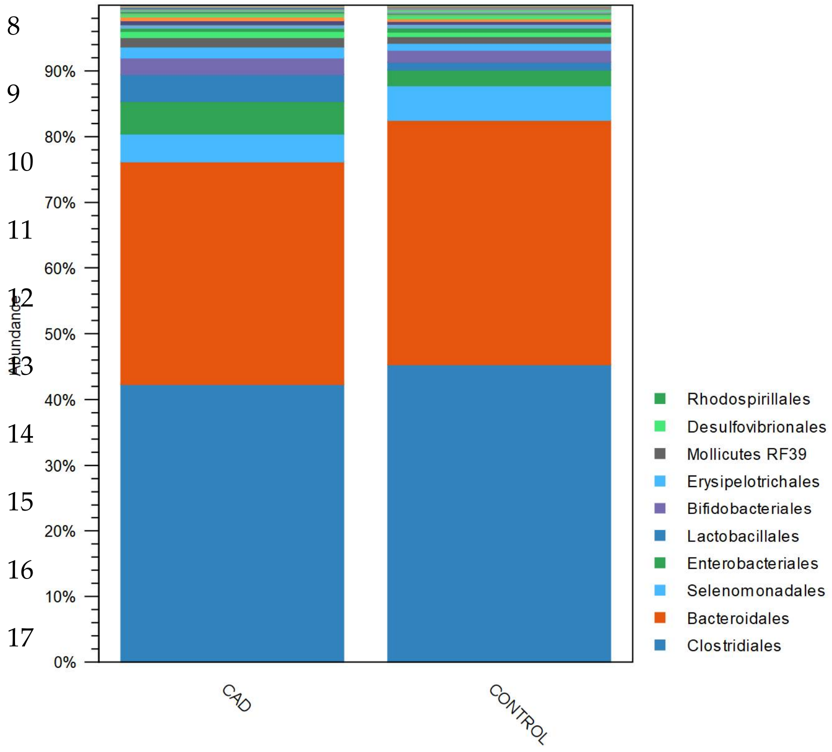

Supplementary Figure S2. Composition of bacteria order in studied populations. CAD – coronary artery disease group.

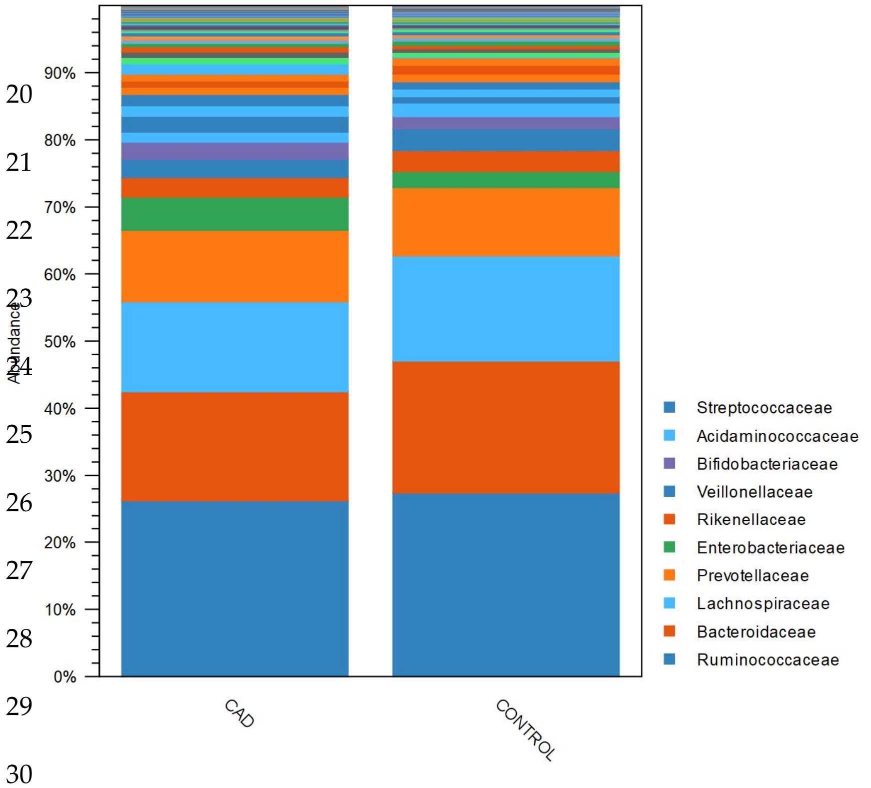

Supplementary Figure S3. Composition of bacteria family in studied populations. CAD – coronary artery disease group.

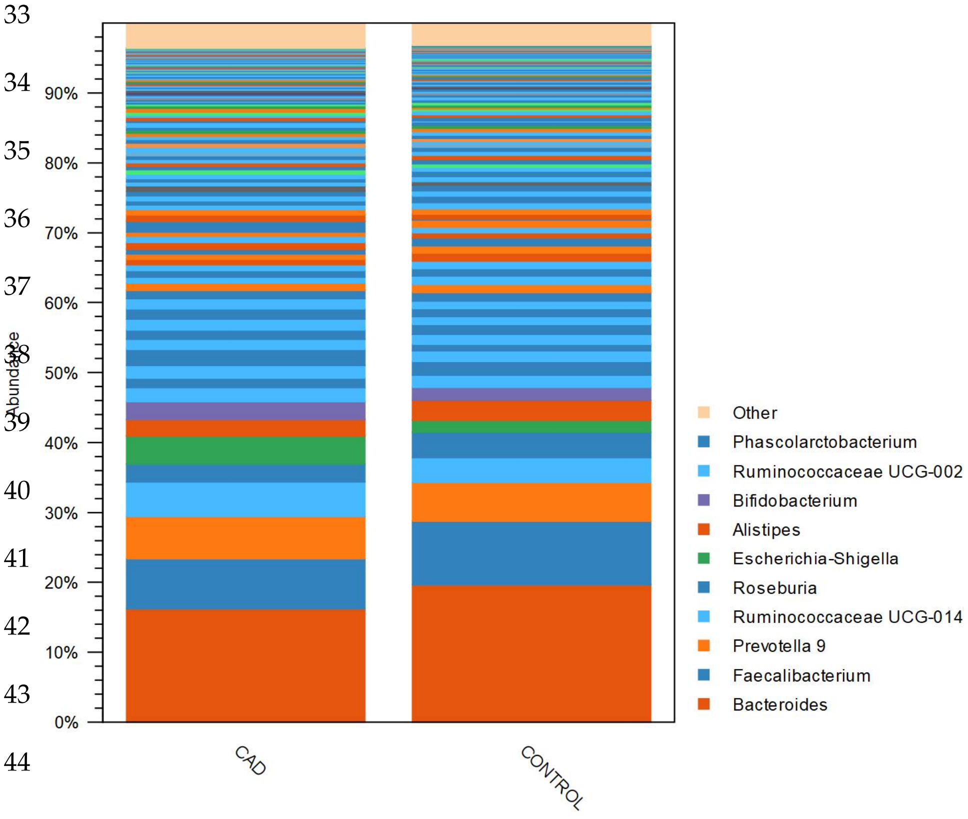

Supplementary Figure S4. Composition of bacteria genus in studied populations. CAD – coronary artery disease group.

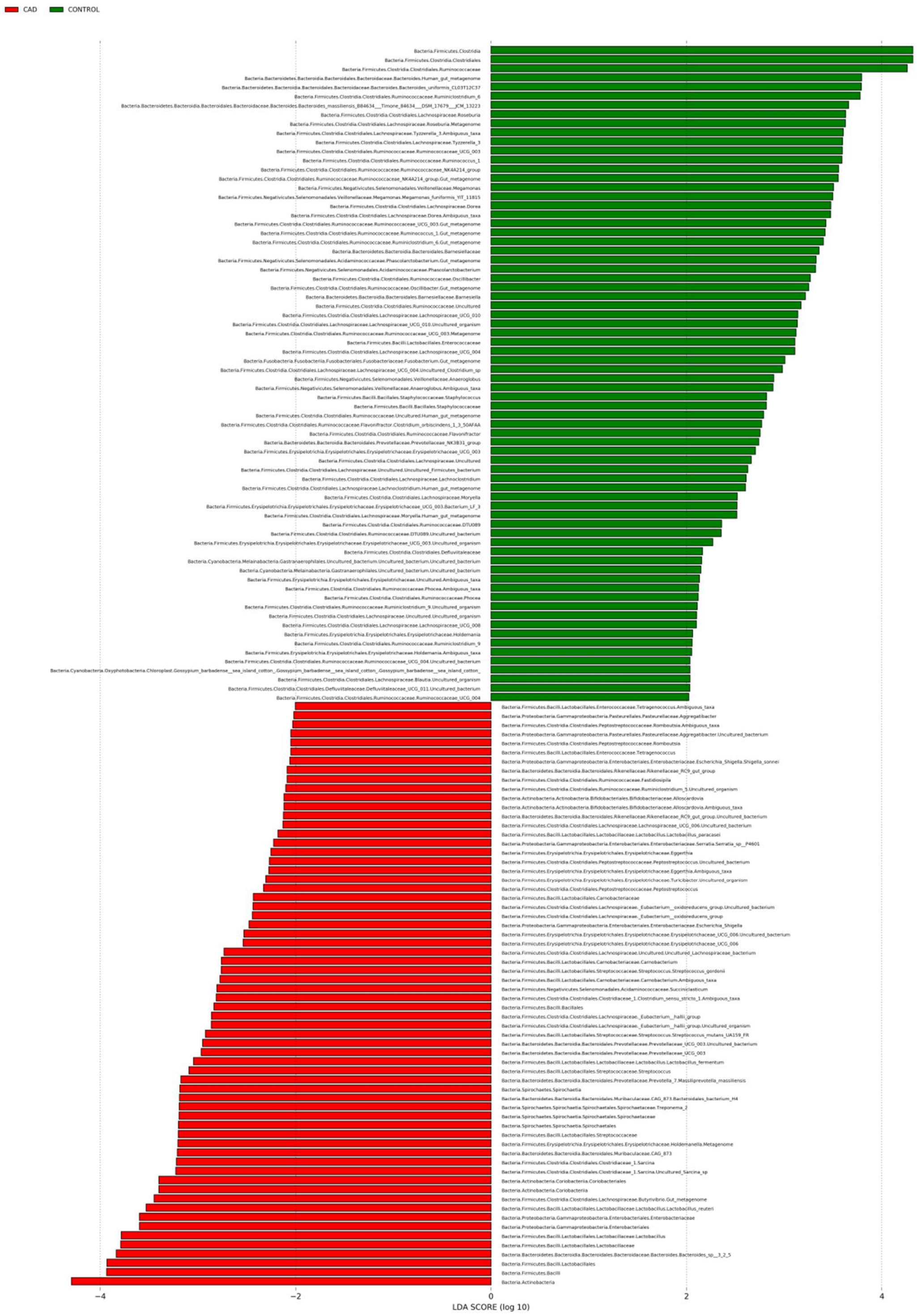

Supplementary Figure S5. Potential biomarkers for coronary artery disease and control group at the taxonomic level. LEfSe identified the major bacteria at all taxonomic levels at the threshold of absolute LDA score 2.

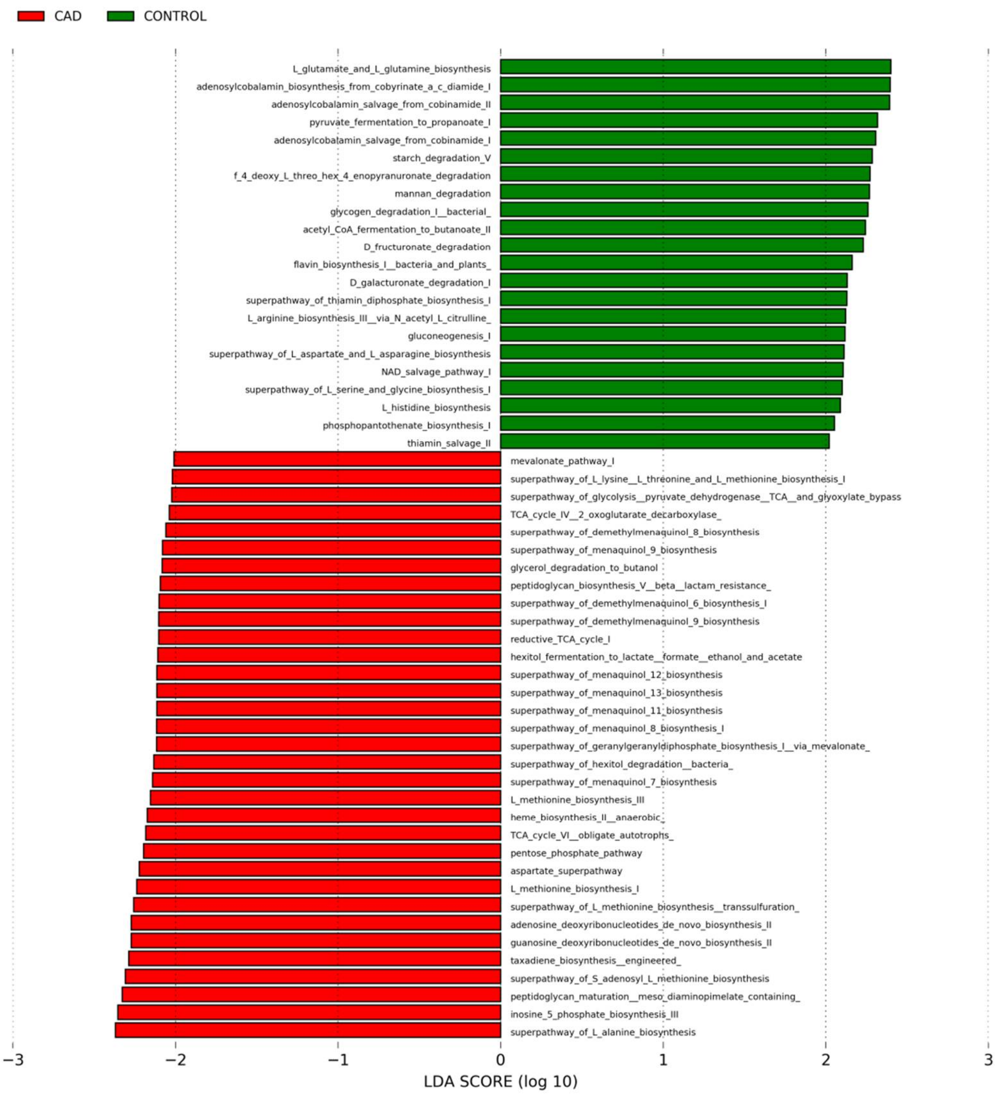

Supplementary Figure S6. Potential biomarkers for coronary artery disease and control group at pathways level. LEfSe identified the major bacteria at all taxonomic levels at the threshold of absolute LDA score 2.

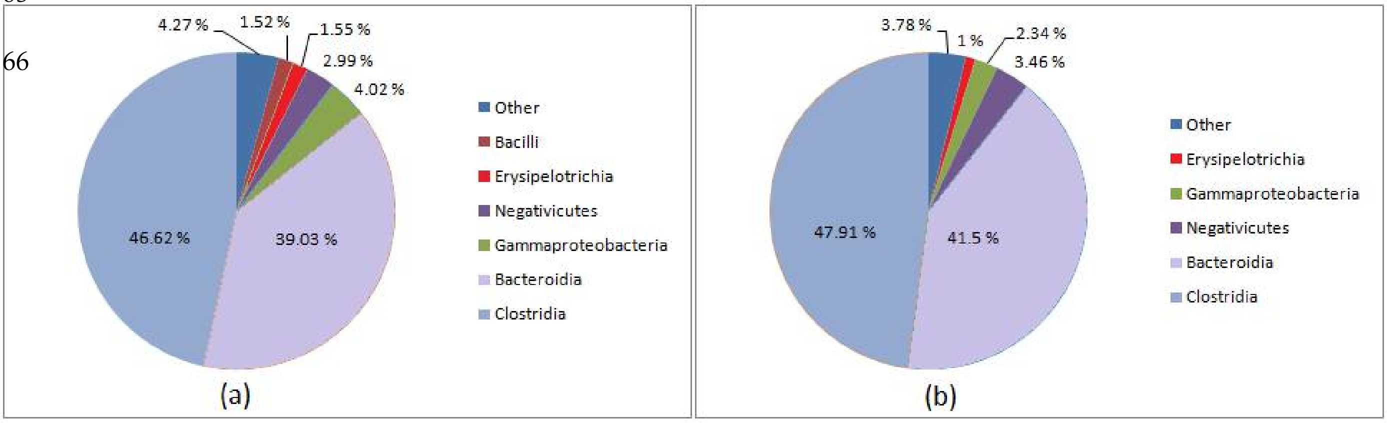

Supplementary Figure S7. Potential DNA topoisomerase producers in coronary artery disease patients (a) and control group (b).

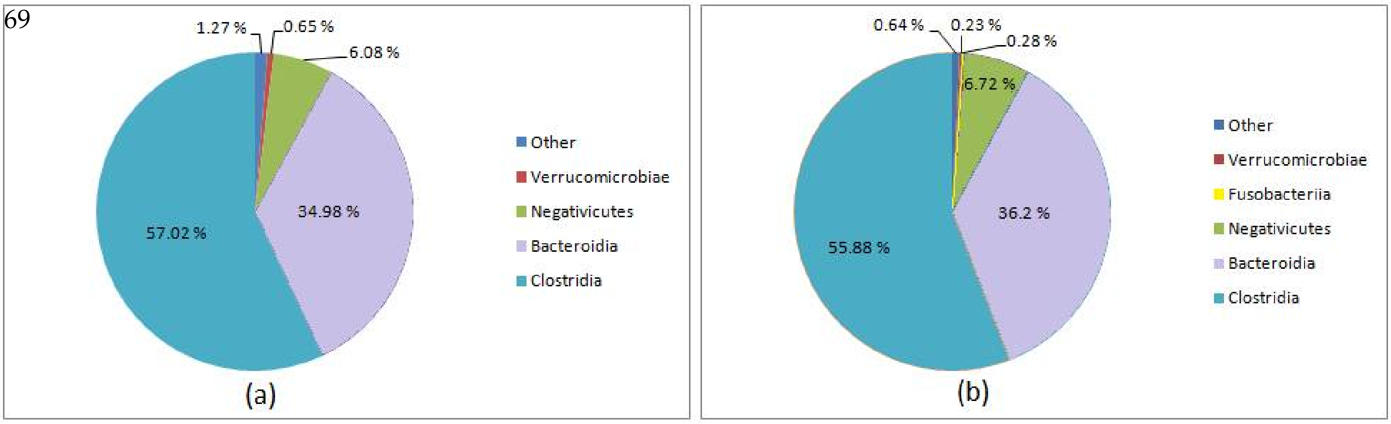

Supplementary Figure S8. Potential oxaloacetate decarboxylase producers in coronary artery disease patients (a) and control group (b).

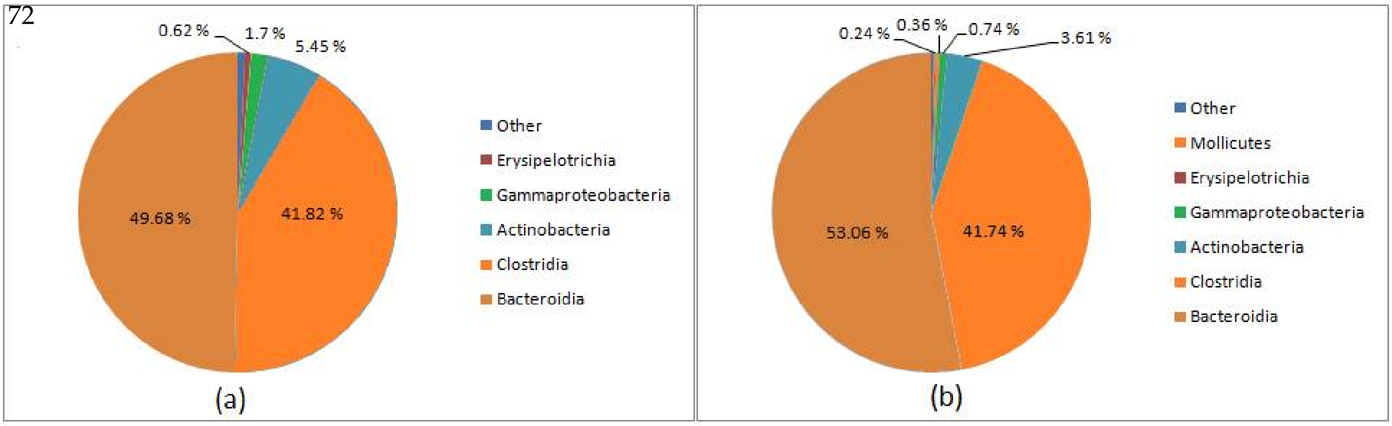

Supplementary Figure S9. Potential beta-glucosidase producers in coronary artery disease patients (a) and control group (b).

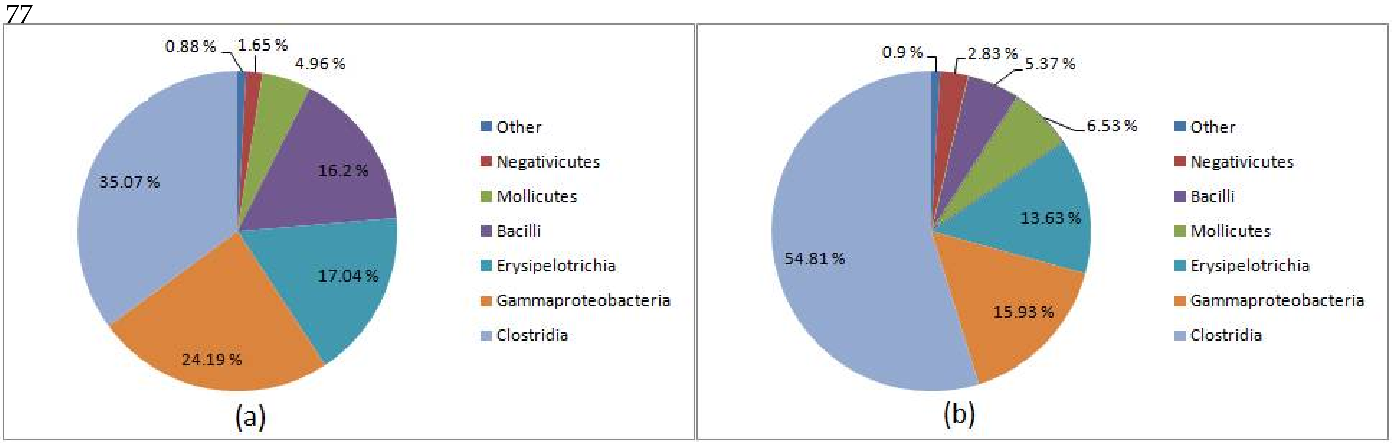

Supplementary Figure S10. Potential 6-phospho-beta-glucosidase producers in coronary artery disease patients (a) and control group (b).

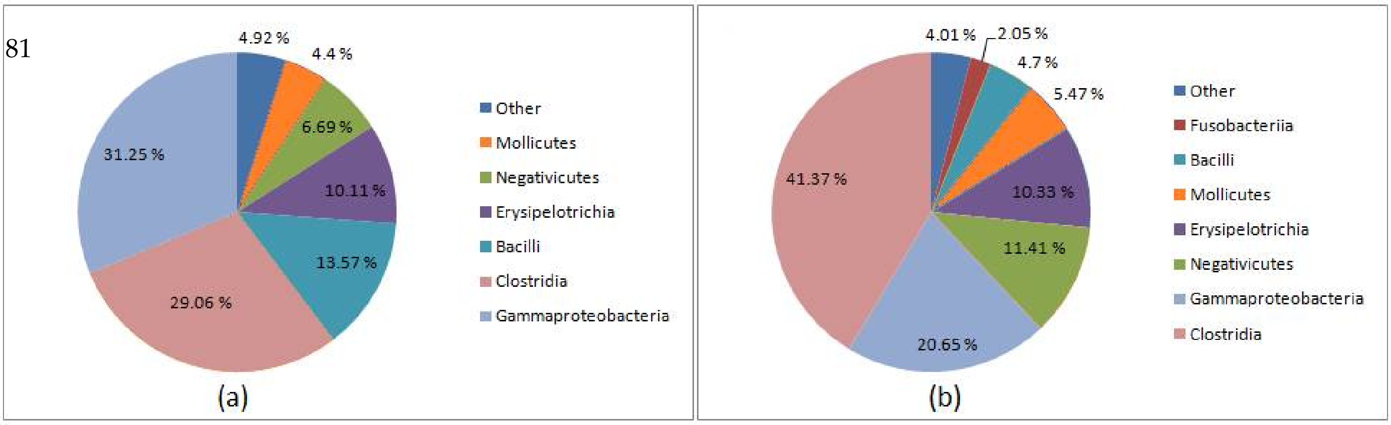

Supplementary Figure S11. Potential protein-N(pi)-phosphohistidine-sugar phosphotransferase producers in coronary artery disease patients (a) and control group (b).

Supplementary Table S1. The differences in relative abundance of bacterial phyla, classes, and orders in studied populations.

**Taxonomic unit**	**Relative abundance in CAD group**	**Relative abundance in control group**	**p-value (adjusted for sex and age)**
**Phyla**			
Firmicutes	52.034 (49.4657 - 54.6023)	52.6381 (50.631 - 54.6452)	0.745
Actinobacteria	3.3228 (2.5724 - 4.0732)	2.4287 (1.8701 - 2.9873)	0.06
Cyanobacteria	0.1242 (0.0571 - 0.1913)	0.2632 (0.1034 - 0.423)	0.111
Spirochaetes	0.0699 (-0.0293 - 0.1691)	0.0012 (-0.0012 - 0.0036)	0.176
Patescibacteria	0.022 (0.0056 - 0.0384)	0.0115 (0.0083 - 0.0147)	0.217
Epsilonbacteraeota	0.0001 (-0.0001 – 0.0003)	0.0002 (0 – 0.0004)	0.255
Verrucomicrobia	1.021 (0.0878 - 1.9542)	0.4623 (0.1554 - 0.7692)	0.264
Synergistetes	0.0153 (0.0022 - 0.0284)	0.0312 (0.0011 - 0.0613)	0.334
Tenericutes	1.4002 (1.0268 - 1.7736)	1.1882 (0.8237 - 1.5527)	0.422
Fusobacteria	0.1591 (0.01 - 0.3082)	0.2249 (-0.0596 - 0.5094)	0.684
Lentisphaerae	0.2813 (0.1928 - 0.3698)	0.2602 (0.1893 - 0.3311)	0.713

Elusimicrobia	0.036 (-0.0343 - 0.1063)	0.0244 (-0.0174 - 0.0662)	0.780
**Classes**			
Actinobacteria_Actinobacteria	2.7165 (2.0288 - 3.4042)	1.9823 (1.4497 - 2.5149)	0.096
Actinobacteria_Coriobacteriia	0.6063 (0.4489 - 0.7637)	0.4464 (0.3298 - 0.563)	0.108
Cyanobacteria_Melainabacteria	0.1103 (0.0442 - 0.1764)	0.2414 (0.0832 - 0.3996)	0.129
Cyanobacteria_Oxyphotobacteria	0.0139 (0.0004 - 0.0274)	0.0219 (0.0003 - 0.0435)	0.533
Elusimicrobia_Elusimicrobia	0.036 (-0.0343 - 0.1063)	0.0244 (-0.0174 - 0.0662)	0.780
Epsilonbacteraeota_Campylobacteria	0.0001 (-0.0001 – 0.0003)	0.0002 (0 – 0.0004)	0.255
Firmicutes_Clostridia	42.387 (39.7322 - 45.0418)	45.174 (42.9875 - 47.3605)	0.110
Firmicutes_Erysipelotrichia	1.4364 (0.919 - 1.9538)	1.0841 (0.7984 - 1.3698)	0.241
Firmicutes_Negativicutes	4.0989 (3.2497 - 4.9481)	5.1593 (4.1275 - 6.1911)	0.117
Fusobacteria_Fusobacteriia	0.1591 (0.01 - 0.3082)	0.2249 (-0.0596 - 0.5094)	0.684
Lentisphaerae_Lentisphaeria	0.2813 (0.1928 - 0.3698)	0.2602 (0.1893 - 0.3311)	0.713
Patescibacteria_Saccharimonadia	0.022 (0.0056 - 0.0384)	0.0115 (0.0083 - 0.0147)	0.217
Proteobacteria_Alphaproteobacteria	0.5106 (0.3007 - 0.7205)	0.5749 (0.407 - 0.7428)	0.637
Proteobacteria_Deltaproteobacteria	0.8772 (0.6808 - 1.0736)	0.6892 (0.5591 - 0.8193)	0.116
Spirochaetes_Brachyspirae	0 (0 - 0)	0.0012 (-0.0012 - 0.0036)	0.314
Spirochaetes_Spirochaetia	0.0699 (-0.0293 - 0.1691)	0 (0 - 0)	0.168
Synergistetes_Synergistia	0.0153 (0.0022 - 0.0284)	0.0312 (0.0011 - 0.0613)	0.334
Tenericutes_Mollicutes	1.4002 (1.0268 - 1.7736)	1.1882 (0.8237 - 1.5527)	0.422
Verrucomicrobia_Verrucomicrobiae	1.021 (0.0878 - 1.9542)	0.4623 (0.1554 - 0.7692)	0.264
**Order**			
Actinobacteria_Actinobacteria_Bifidobacteriales	2.6688 (1.9835 - 3.3541)	1.9585 (1.4261 - 2.4909)	0.107
Actinobacteria_Actinobacteria_Corynebacteriales	0.0003 (0.0001 - 0.0005)	0.0003 (0.0001 - 0.0005)	0.911
Actinobacteria_Actinobacteria_Propionibacteriales	0.0042 (0.0004 - 0.008)	0.0017 (0.0005 - 0.0029)	0.197
Actinobacteria_Coriobacteriia_Coriobacteriales	0.6063 (0.4489 - 0.7637)	0.4464 (0.3298 - 0.563)	0.108
Bacteroidetes_Bacteroidia_Flavobacteriales	0.062 (0.0378 - 0.0862)	0.0727 (0.043 - 0.1024)	0.578
Cyanobacteria_Melainabacteria_Gastranaerophilales	0.1103 (0.0442 - 0.1764)	0.2414 (0.0832 - 0.3996)	0.129
Cyanobacteria_Oxyphotobacteria_Chloroplast	0.0139 (0.0004 - 0.0274)	0.0219 (0.0003 - 0.0435)	0.533
Elusimicrobia_Elusimicrobia_Elusimicrobiales	0.036 (-0.0343 - 0.1063)	0.0244 (-0.0174 - 0.0662)	0.780
Epsilonbacteraeota_Campylobacteria_Campylobacterales	0.0001 (-0.0001 - 0.0004)	0.0002 (0 - 0.0004)	0.255
Firmicutes_Bacilli_Bacillales	0.2207 (0.0492 - 0.3922)	0.0568 (0.0077 - 0.1059)	0.072
Firmicutes_Clostridia_Clostridiales	42.3791 (39.7249 - 45.0333)	45.1646 (42.9787 - 47.3505)	0.110
Firmicutes_Clostridia_DTU014	0.0079 (0.0051 - 0.0107)	0.0094 (0.0064 - 0.0124)	0.474
Firmicutes_Erysipelotrichia_Erysipelotrichales	1.4364 (0.919 - 1.9538)	1.0841 (0.7984 - 1.3698)	0.241
Firmicutes_Negativicutes_Selenomonadales	4.0989 (3.2497 - 4.9481)	5.1593 (4.1275 - 6.1911)	0.117
Fusobacteria_Fusobacteriia_Fusobacteriales	0.1591 (0.01 - 0.3082)	0.2249 (-0.0596 - 0.5094)	0.684
Lentisphaerae_Lentisphaeria_Victivallales	0.2813 (0.1928 - 0.3698)	0.2602 (0.1893 - 0.3311)	0.713
Patescibacteria_Saccharimonadia_Saccharimonadales	0.022 (0.0056 - 0.0384)	0.0115 (0.0083 - 0.0147)	0.217
Proteobacteria_Alphaproteobacteria_Rhizobiales	0.0029 (-0.0001 - 0.0059)	0.0009 (-0.0001 - 0.0019)	0.219
Proteobacteria_Alphaproteobacteria_Rhodospirillales	0.5074 (0.2975 - 0.7173)	0.5715 (0.4038 - 0.7392)	0.638
Proteobacteria_Alphaproteobacteria_Rickettsiales	0.0004 (-0.0002 - 0.001)	0.0025 (-0.0011 - 0.0061)	0.234
Proteobacteria_Deltaproteobacteria_Desulfovibrionales	0.8772 (0.6808 - 1.0736)	0.6892 (0.5591 - 0.8193)	0.116
Proteobacteria_Gammaproteobacteria_Aeromonadales	0.1592 (0.0147 - 0.3037)	0.3233 (0.0669 - 0.5797)	0.268
Proteobacteria_Gammaproteobacteria_Alteromonadales	0.0007 (-0.0003 - 0.0017)	0.0044 (-0.0039 - 0.0127)	0.38
Proteobacteria_Gammaproteobacteria_Betaproteobacteriales	0.2573 (0.144 - 0.3706)	0.3721 (0.2297 - 0.5145)	0.21
Proteobacteria_Gammaproteobacteria_Pasteurellales	0.2048 (0.1018 - 0.3078)	0.1502 (0.0722 - 0.2282)	0.405
Proteobacteria_Gammaproteobacteria_Pseudomonadales	0.0004 (0 - 0.0008)	0.0016 (0.0008 - 0.004)	0.303

Proteobacteria_Gammaproteobacteria_Vibrionales	0.0004 (0 - 0.0008)	0.0002 (0 - 0.0004)	0.119
Spirochaetes_Brachyspirae_Brachyspirales	0 (0 - 0)	0.0012 (-0.0012 - 0.0036)	0.314
Spirochaetes_Spirochaetia_Spirochaetales	0.0699 (-0.0293 - 0.1691)	0 (0 - 0)	0.168
Synergistetes_Synergistia_Synergistales	0.0153 (0.0022 - 0.0284)	0.0312 (0.0011 - 0.0613)	0.334
Tenericutes_Mollicutes_Anaeroplasmatales	0 (0 - 0)	0.0002 (0 - 0.0004)	0.244
Tenericutes_Mollicutes_Izimaplasmatales	0.1066 (0.0529 - 0.1603)	0.1957 (0.0636 - 0.3278)	0.214
Tenericutes_Mollicutes_Mollicutes.RF39	1.2936 (0.9305 - 1.6567)	0.9923 (0.6832 - 1.3014)	0.212
Verrucomicrobia_Verrucomicrobiae_Opitutales	0 (0 - 0)	0.0087 (-0.0083 - 0.0257)	0.312
Verrucomicrobia_Verrucomicrobiae_Verrucomicrobiales	1.021 (0.0878 - 1.9542)	0.4536 (0.1469 - 0.7603)	0.257

CAD – coronary artery disease group, CI - confidence interval. Mean values together with the 95% confidence intervals of the means are presented.

Supplementary Table S2. Statistically significant differences in the relative abundance of bacterial families and genera in studied populations.

**Taxonomic unit**	**Relative abundance in CAD group**	**Relative abundance in control group**	**p (adjusted for sex and age)**
**Families**			
Actinobacteria_Actinobacteria_Actinomycetales_Actinomycetaceae	0.0281 (0.0164 - 0.0398)	0.0135 (0.0095 - 0.0175)	*0.021*
Actinobacteria_Actinobacteria_Micrococcales_Micrococcaceae	0.0152 (0.0108 - 0.0196)	0.0083 (0.0043 - 0.0123)	*0.021*
Actinobacteria_Coriobacteriia_Coriobacteriales_Atopobiaceae	0.0499 (0.0269 - 0.0729)	0.0175 (0.0096 - 0.0254)	*0.009*
Bacteroidetes_Bacteroidia_Bacteroidales_Barnesiellaceae	0.8394 (0.6557 - 1.0231)	1.2015 (0.9815 - 1.4215)	*0.013*
Firmicutes_Bacilli_Lactobacillales_Lactobacillaceae	1.4705 (0.751 - 2.19)	0.1491 (0.0828 - 0.2154)	*<0.001*
Firmicutes_Bacilli_Lactobacillales_Streptococcaceae	2.2671 (1.4527 - 3.0815)	0.9256 (0.5338 - 1.3174)	*0.004*
Proteobacteria_Gammaproteobacteria_Enterobacteriales_Enterobacteriaceae	5.4589 (3.1754 - 7.7424)	2.5555 (1.4063 - 3.7047)	*0.026*
**Genera**			
Actinobacteria_Actinobacteria_Actinomycetales_Actinomycetaceae_Actinomyces	0.028 (0.0163 - 0.0397)	0.0133 (0.0093 - 0.0173)	*0.020*
Actinobacteria_Actinobacteria_Bifidobacteriales_Bifidobacteriaceae_Alloscardovia	0.0045 (0.0013 - 0.0077)	0.0007 (0.0003 - 0.0011)	*0.025*
Actinobacteria_Actinobacteria_Micrococcales_Micrococcaceae_Rothia	0.0152 (0.0108 - 0.0196)	0.0083 (0.0043 - 0.0123)	*0.021*
Actinobacteria_Coriobacteriia_Coriobacteriales_Atopobiaceae_Atopobium	0.006 (0.0034 - 0.0086)	0.0021 (0.0013 - 0.0029)	*0.003*
Bacteroidetes_Bacteroidia_Bacteroidales_Barnesiellaceae_Barnesiella	0.7082 (0.5405 - 0.8759)	1.035 (0.822 - 1.248)	*0.017*
Bacteroidetes_Bacteroidia_Bacteroidales_Barnesiellaceae_Coprobacter	0.0766 (0.0554 - 0.0978)	0.1114 (0.0849 - 0.1379)	*0.044*
Bacteroidetes_Bacteroidia_Bacteroidales_Prevotellaceae_Paraprevotella	0.559 (0.4048 - 0.7132)	0.9423 (0.6885 - 1.1961)	*0.011*
Firmicutes_Bacilli_Lactobacillales_Carnobacteriaceae_Carnobacterium	0.0003 (0.0001 – 0.0005)	(0 - 0)	*0.049*
Firmicutes_Bacilli_Lactobacillales_Lactobacillaceae_Lactobacillus	1.4644 (0.7449 - 2.1839)	0.1461 (0.0806 - 0.2116)	*<0.001*
Firmicutes_Bacilli_Lactobacillales_Streptococcaceae_Streptococcus	2.2143 (1.4065 - 3.0221)	0.9119 (0.5201 - 1.3037)	*0.005*
Firmicutes_Clostridia_Clostridiales_Clostridiales.vadinBB60.group_Ambiguous	0.1537 (0.089 - 0.2184)	0.3323 (0.1832 - 0.4814)	*0.029*

Firmicutes_Clostridia_Clostridiales_Family.XI_Peptoniphilus	0.003 (0.0008 - 0.0052)	0.0006 (0 - 0.0012)	*0.036*
Firmicutes_Clostridia_Clostridiales_Family.XIII_Eubacterium.nodatum.group	0.0059 (0.0033 - 0.0085)	0.0108 (0.0066 - 0.015)	*0.046*
Firmicutes_Clostridia_Clostridiales_Lachnospiraceae_Coprococcus.3	0.0007 (0.0001 - 0.0013)	0.002 (0.0014 - 0.0026)	*0.013*
Firmicutes_Clostridia_Clostridiales_Lachnospiraceae_Eubacterium.hallii.group	0.0453 (0.0217 - 0.0689)	0.019 (0.0075 - 0.0305)	*0.049*
Firmicutes_Clostridia_Clostridiales_Lachnospiraceae_GCA.900066755	0.001 (0.0006 - 0.0014)	0.0025 (0.0017 - 0.0033)	*0.002*
Firmicutes_Clostridia_Clostridiales_Lachnospiraceae_Lachnospiraceae.NK4A136.group	0.525 (0.365 - 0.685)	0.9508 (0.7558 - 1.1458)	*<0.001*
Firmicutes_Clostridia_Clostridiales_Lachnospiraceae_Lachnospiraceae.UCG.006	0.1738 (0.1213 - 0.2263)	0.2881 (0.234 - 0.3422)	*0.003*
Firmicutes_Clostridia_Clostridiales_Lachnospiraceae_Lachnospiraceae.UCG.008	0.4953 (0.4086 - 0.582)	0.7278 (0.6021 - 0.8535)	*0.003*
Firmicutes_Clostridia_Clostridiales_Lachnospiraceae_Lachnospiraceae.UCG.010	0.1136 (0.077 - 0.1502)	0.2182 (0.1691 - 0.2673)	*<0.001*
Firmicutes_Clostridia_Clostridiales_Lachnospiraceae_Roseburia	2.7577 (2.3439 - 3.1715)	3.3971 (2.9286 - 3.8656)	*0.043*
Firmicutes_Clostridia_Clostridiales_Lachnospiraceae_Tyzzerella.3	0.1266 (0.064 - 0.1892)	0.3635 (0.2146 - 0.5124)	*0.004*
Firmicutes_Clostridia_Clostridiales_Lachnospiraceae_Uncultured	0.5817 (0.4714 - 0.692)	0.8661 (0.7578 - 0.9744)	*<0.001*
Firmicutes_Clostridia_Clostridiales_Peptococcaceae_Uncultured	0.0182 (0.0136 - 0.0228)	0.0377 (0.0264 - 0.049)	*0.002*
Firmicutes_Clostridia_Clostridiales_Peptostreptococcaceae_Peptostreptococcus	0.0003 (0.0001 – 0.0005)	(0 - 0)	*0.049*
Firmicutes_Clostridia_Clostridiales_Ruminococcaceae_Ambiguous	0.0161 (0.0098 - 0.0224)	0.028 (0.0217 - 0.0343)	*0.01*
Firmicutes_Clostridia_Clostridiales_Ruminococcaceae_Anaerofilum	0.0039 (0.0027 - 0.0051)	0.0074 (0.0058 - 0.009)	*<0.001*
Firmicutes_Clostridia_Clostridiales_Ruminococcaceae_Anaerotruncus	0.01 (0.0062 - 0.0138)	0.0168 (0.0115 - 0.0221)	*0.038*
Firmicutes_Clostridia_Clostridiales_Ruminococcaceae_Butyricicoccus	0.2359 (0.191 - 0.2808)	0.3172 (0.2568 - 0.3776)	*0.033*
Firmicutes_Clostridia_Clostridiales_Ruminococcaceae_DTU089	0.0101 (0.0061 - 0.0141)	0.0203 (0.0152 - 0.0254)	*0.002*
Firmicutes_Clostridia_Clostridiales_Ruminococcaceae_Faecalibacterium	7.1013 (6.2404 - 7.9622)	8.8906 (8.1166 - 9.6646)	*0.002*
Firmicutes_Clostridia_Clostridiales_Ruminococcaceae_Flavonifractor	0.0521 (0.0325 - 0.0717)	0.0855 (0.0608 - 0.1103)	*0.036*
Firmicutes_Clostridia_Clostridiales_Ruminococcaceae_Harryflintia	0.0013 (0.0009 - 0.0017)	0.0028 (0.0014 - 0.0042)	*0.039*
Firmicutes_Clostridia_Clostridiales_Ruminococcaceae_Intestinimonas	0.0186 (0.0121 - 0.0251)	0.0356 (0.0198 - 0.0514)	*0.049*
Firmicutes_Clostridia_Clostridiales_Ruminococcaceae_Oscillibacter	0.1351 (0.0981 - 0.1721)	0.2792 (0.1978 - 0.3606)	*0.001*
Firmicutes_Clostridia_Clostridiales_Ruminococcaceae_Phocea	0.004 (0.0024 - 0.0056)	0.0089 (0.0065 - 0.0113)	*<0.001*
Firmicutes_Clostridia_Clostridiales_Ruminococcaceae_Ruminiclostridium	0.0116 (0.008 - 0.0152)	0.0201 (0.0148 - 0.0254)	*0.009*
Firmicutes_Clostridia_Clostridiales_Ruminococcaceae_Ruminiclostridium.6	0.2491 (0.1648 - 0.3334)	0.5274 (0.404 - 0.6508)	*<0.001*
Firmicutes_Clostridia_Clostridiales_Ruminococcaceae_Ruminococcaceae.UCG.009	0.0153 (0.0109 - 0.0197)	0.0243 (0.0186 - 0.03)	*0.013*
Firmicutes_Clostridia_Clostridiales_Ruminococcaceae_Ruminococcus.1	0.8531 (0.7092 - 0.997)	1.2645 (1.0556 - 1.4734)	*0.001*

Firmicutes_Clostridia_Clostridiales_Ruminococcaceae_Uncultured	0.7571 (0.6272 - 0.887)	1.0365 (0.887 - 1.186)	*0.005*
Firmicutes_Erysipelotrichia_Erysipelotrichales_Erysipelotrichaceae_Holdemania	0.0071 (0.0051 - 0.0091)	0.0152 (0.011 - 0.0194)	*<0.001*
Proteobacteria_Alphaproteobacteria_Rhodospirillales_Uncultured_Uncultured.bacterium	0.0109 (0.0004 - 0.0214)	0.0715 (0.0151 - 0.1279)	*0.036*

CAD – coronary artery disease group, CI - confidence interval. Mean values together with the 95% confidence intervals of the means are presented.

Supplementary Table S3. The influence of Firmicutes/Bacteroidetes ratio on targeted metabolomics (Biocrates) and biochemical test adjusted for age, sex, CAD status, statins treatment (and) LDL cholesterol concentration.

Metabolite, µmol/L	Group with F/B ratio ≤ 1.54 (n = 167)	Group with F/B ratio > 1.54 (n = 167)	P value (* adjusted for age, sex, CAD status, LDL cholesterol concentration, statins treatment ** adjusted for age, sex, CAD status, statins treatment)
Acylcarnitines C0	6228.025 (95% CI 5865.0888 - 6590.9612)	6442.4714 (95% CI 6050.7082 - 6834.2346)	0.394195
Acylcarnitines C10	91.9532 (95% CI 84.6052 - 99.3012)	104.1058 (95% CI 80.2349 - 127.9767)	0.310319
Acylcarnitines C12	45.0194 (95% CI 41.1505 - 48.8883)	58.4254 (95% CI 30.2225 - 86.6283)	0.333143
Acylcarnitines C14.1	56.8537 (95% CI 47.2186 - 66.4888)	52.9099 (95% CI 41.598 - 64.2218)	0.591831
Acylcarnitines C14.1.OH	16.0353 (95% CI 0.3343 - 31.7363)	78.7392 (95% CI -16.6523 - 174.1307)	0.201986
Acylcarnitines C14.2	32.3733 (95% CI 7.9631 - 56.7835)	53.3412 (95% CI 0.2622 - 106.4202)	0.538153
Acylcarnitines C16	62.5412 (95% CI 53.3689 - 71.7136)	95.8507 (95% CI 38.7063 - 152.9951)	0.232551
Acylcarnitines C16.1	23.6724 (95% CI 17.4194 - 29.9254)	38.0786 (95% CI 10.002 - 66.1552)	0.317191
Acylcarnitines C18	29.8955 (95% CI 21.5163 - 38.2747)	110.0393 (95% CI -49.4941 - 269.5727)	0.288166
Acylcarnitines C18.1	76.9662 (95% CI 69.0298 - 84.9026)	110.307 (95% CI 33.2622 - 187.3518)	0.36986
Acylcarnitines C18.2	31.2203 (95% CI 17.3391 - 45.1015)	71.8563 (95% CI -20.2222 - 163.9348)	0.369156
Acylcarnitines C2	1660.6125 (95% CI 1530.1463 - 1791.0787)	1532.2535 (95% CI 1404.6255 - 1659.8815)	0.168835
Acylcarnitines C3	84.4912 (95% CI 77.5442 - 91.4382)	82.3549 (95% CI 76.6111 - 88.0987)	0.658476
Acylcarnitines C3.1	8.1423 (95% CI -1.8246 - 18.1092)	20.2436 (95% CI -9.0578 - 49.545)	0.452543
Acylcarnitines C4	52.15 (95% CI 47.3346 - 56.9654)	56.2366 (95% CI 47.3424 - 65.1308)	0.408403
Acylcarnitines C4.1	14.1714 (95% CI 6.8735 - 21.4693)	5.0609 (95% CI 1.5167 - 8.6051)	0.053336

Acylcarnitines C5	42.7348 (95% CI 38.9288 - 46.5408)	44.618 (95% CI 41.4494 - 47.7866)	0.401277
Acylcarnitines C5.1.DC	8.948 (95% CI 4.9823 - 12.9137)	8.1004 (95% CI 5.9949 - 10.2059)	0.751624
Acylcarnitines C5.DC.C6.OH.	14.5848 (95% CI 8.3933 - 20.7763)	17.1242 (95% CI 8.7282 - 25.5202)	0.643354
Acylcarnitines C5.M.DC	15.48 (95% CI 11.9433 - 19.0167)	16.3322 (95% CI 11.2691 - 21.3953)	0.731803
Acylcarnitines C5.OH.C3.DC.M.	13.293 (95% CI 10.089 - 16.497)	13.26 (95% CI 10.9398 - 15.5802)	0.932428
Acylcarnitines C6.1	17.0942 (95% CI 1.4207 - 32.7677)	15.2214 (95% CI -3.2898 - 33.7326)	0.885145
Acylcarnitines C6.C4.1.DC.	30.4615 (95% CI 26.51 - 34.413)	45.3963 (95% CI 14.5008 - 76.2918)	0.410314
Acylcarnitines C7.DC	26.6438 (95% CI 18.7446 - 34.543)	40.4308 (95% CI 5.2973 - 75.5643)	0.425139
Acylcarnitines C8	69.7018 (95% CI 64.8356 - 74.568)	77.6435 (95% CI 68.3977 - 86.8893)	0.119552
Acylcarnitines C9	36.6891 (95% CI -9.3659 - 82.7441)	122.4595 (95% CI -71.3738 - 316.2928)	0.546283
Aminoacids Ala	38552.325 (95% CI 36194.2236 - 40910.4264)	813246.2394 (95% CI -723091.6358 - 2349584.1146)	0.294469
Aminoacids Arg	19561.275 (95% CI 18757.6407 - 20364.9093)	19592.9718 (95% CI 18686.6783 - 20499.2653)	0.951491
Aminoacids Asn	6243.8125 (95% CI 5950.2195 - 6537.4055)	18882.8873 (95% CI -6332.742 - 44098.5166)	0.2973
Aminoacids Asp	3483.7051 (95% CI 3199.2755 - 3768.1347)	32823.5942 (95% CI -25708.5615 - 91355.7499)	0.296448
Aminoacids Cit	5754.8375 (95% CI 5399.2632 - 6110.4118)	64159.0563 (95% CI -51228.1535 - 179546.2661)	0.29263
Aminoacids Gln	107208.0875 (95% CI 103710.8375 - 110705.3375)	109585.3521 (95% CI 105921.7866 - 113248.9176)	0.342918
Aminoacids Glu	11749.5584 (95% CI 10619.8854 - 12879.2314)	200781.3971 (95% CI -174099.778 - 575662.5722)	0.293221
Aminoacids Gly	20471.7375 (95% CI 19020.568 - 21922.907)	18799.3099 (95% CI 17609.0424 - 19989.5774)	0.086364
Aminoacids His	14484.2125 (95% CI 14016.6876 - 14951.7374)	14794.2113 (95% CI 14296.2985 - 15292.1241)	0.351484
Aminoacids Ile	11749.5625 (95% CI 11028.1554 - 12470.9696)	11978.1549 (95% CI 11294.5753 - 12661.7345)	0.598171
Aminoacids Leu	24346.975 (95% CI 23061.681 - 25632.269)	24764.5634 (95% CI 23607.3233 - 25921.8035)	0.580081
Aminoacids Lys	36647.325 (95% CI 35187.3209 - 38107.3291)	38373.8451 (95% CI 36626.601 - 40121.0892)	0.118937
Aminoacids Met	3705.2857 (95% CI 3535.2661 - 3875.3053)	3833.4923 (95% CI 3641.3188 - 4025.6658)	0.29102
Aminoacids Orn	12454.225 (95% CI 11656.6281 - 13251.8219)	12974.3803 (95% CI 12052.7471 - 13896.0135)	0.304364
Aminoacids Phe	12947.5625 (95% CI 12362.4412 - 13532.6838)	12773.8028 (95% CI 12231.2636 - 13316.342)	0.692633
Aminoacids Pro	20963.35 (95% CI 19672.5959 - 22254.1041)	22298.169 (95% CI 20728.8933 - 23867.4447)	0.154627
Aminoacids Ser	15089.95 (95% CI 14425.9148 - 15753.9852)	14558.4507 (95% CI 13963.228 - 15153.6733)	0.245737
Aminoacids Thr	14448.2 (95% CI 13723.6285 - 15172.7715)	181938.4507 (95% CI -149073.5731 - 512950.4745)	0.292781
Aminoacids Trp	13913.625	14120.7606 (95% CI 13627.7602 -	0.501583

	(95% CI 13432.9182 - 14394.3318)	14613.761)	
Aminoacids Tyr	14134.9625 (95% CI 13441.3327 - 14828.5923)	14351.3803 (95% CI 13635.0555 - 15067.7051)	0.59819
Aminoacids Val	32503.6875 (95% CI 30915.7758 - 34091.5992)	33617.507 (95% CI 32035.7369 - 35199.2771)	0.297339
Biogenic.Amines Ac.Orn	50.9478 (95% CI 50.6051 - 51.2905)	50.7077 (95% CI 50.4968 - 50.9186)	0.565192
Biogenic.Amines ADMA	109.414 (95% CI 103.2287 - 115.5993	3453.5489 (95% CI -3168.2683 - 10075.3661)	0.307849
Biogenic.Amines alpha.AAA	224.2831 (95% CI 205.3335 - 243.2327)	218.0236 (95% CI 198.7948 - 237.2524)	0.6967
Biogenic.Amines c4.OH.Pro	8.0352 (95% CI 7.8356 - 8.2348)	7.9012 (95% CI 7.8075 - 7.9949)	0.391707
Biogenic.Amines Carnosine	34.0396 (95% CI 28.0097 - 40.0695)	38.5341 (95% CI 32.388 - 44.6802)	0.332668
Biogenic.Amines Creatinine	9456.5844 (95% CI 9014.3924 - 9898.7764)	112214.4925 (95% CI -91269.5955 - 315698.5805)	0.288438
Biogenic.Amines DOPA	51.3125 (95% CI 50.4732 - 52.1518)	90.0511 (95% CI 12.8719 - 167.2303)	0.323331
Biogenic.Amines Dopamine	67.3792 (95% CI 59.4067 - 75.3517)	72.5978 (95% CI 64.3976 - 80.798)	0.401899
Biogenic.Amines Histamine	48.1688 (95% CI 40.8147 - 55.5229)	53.7341 (95% CI 46.1994 - 61.2688)	0.326598
Biogenic.Amines Kynurenine	658.0274 (95% CI 625.0558 - 690.999)	670.0156 (95% CI 634.479 - 705.5522)	0.564147
Biogenic.Amines Met.SO	90.6043 (95% CI 78.6099 - 102.5987)	93.4455 (95% CI 82.1894 - 104.7016)	0.599824
Biogenic.Amines Nitro.Tyr	53.8042 (95% CI 46.2891 - 61.3193)	59.8364 (95% CI 52.2025 - 67.4703)	0.291585
Biogenic.Amines Putrescine	16.4222 (95% CI 15.1851 - 17.6593)	151.9271 (95% CI -115.8148 - 419.669)	0.287407
Biogenic.Amines Sarcosine	157.7667 (95% CI 141.4563 - 174.0771)	2786.28 (95% CI -2437.9506 - 8010.5106)	0.303657
Biogenic.Amines SDMA	124.9559 (95% CI 117.3937 - 132.5181)	3881.7786 (95% CI -3555.9774 - 11319.5346)	0.278513
Biogenic.Amines Serotonin	108.2559 (95% CI 91.9847 - 124.5271)	1733.7544 (95% CI -1505.4068 - 4972.9156)	0.282177
Biogenic.Amines Spermidine	34.1208 (95% CI 31.7143 - 36.5273)	58.2422 (95% CI 14.8909 - 101.5935)	0.270366
Biogenic.Amines Spermine	32.1 (95% CI 28.0818 - 36.1182)	39.4542 (95% CI 24.5973 - 54.3111)	0.47012
Biogenic.Amines t4.OH.Pro	1655.9125 (95% CI 1458.0555 - 1853.7695)	1704.3521 (95% CI 1513.3647 - 1895.3395)	0.713953
Biogenic.Amines Taurine	16431.5375 (95% CI 15653.9588 - 17209.1162)	18388.9296 (95% CI 14519.3124 - 22258.5468)	0.298395
Glycerophospholipids lysoPC.a.C14.0	2358.4625 (95% CI 2040.1591 - 2676.7659)	2334.9577 (95% CI 2066.2628 - 2603.6526)	0.91158
Glycerophospholipids lysoPC.a.C16.0	40229.875 (95% CI 37904.5612 - 42555.1888)	38141.7465 (95% CI 35774.3484 - 40509.1446)	0.220651
Glycerophospholipids lysoPC.a.C16.1	1138.8375 (95% CI 1045.7929 - 1231.8821)	1142 (95% CI 924.8712 - 1359.1288)	0.970598
Glycerophospholipids lysoPC.a.C17.0	688.675 (95% CI 571.0064 - 806.3436)	778.7465 (95% CI 535.399 - 1022.094)	0.499013
Glycerophospholipids lysoPC.a.C18.0	13684.75 (95% CI 12887.7295 - 14481.7705)	13559.1549 (95% CI 12094.8194 - 15023.4904)	0.88543
Glycerophospholipids lysoPC.a.C18.1	9342.2375 (95% CI 8662.4885 - 10021.9865)	8919.6197 (95% CI 8323.4217 - 9515.8177)	0.366214

Glycerophospholipids lysoPC.a.C18.2	11223.5625 (95% CI 10340.8199 - 12106.3051)	11336.8732 (95% CI 10414.7159 - 12259.0305)	0.872987
Glycerophospholipids lysoPC.a.C20.3	936.8228 (95% CI 873.0539 - 1000.5917)	950.9296 (95% CI 863.1235 - 1038.7357)	0.790362
Glycerophospholipids lysoPC.a.C20.4	3228.15 (95% CI 2865.5894 - 3590.7106)	2971.0845 (95% CI 2708.6008 - 3233.5682)	0.273605
Glycerophospholipids lysoPC.a.C24.0	1297.4 (95% CI -933.8036 - 3528.6036)	9282 (95% CI -7620.1207 - 26184.1207)	0.171411
Glycerophospholipids lysoPC.a.C26.1	269.9651 (95% CI 18.7395 - 521.1907)	666.8808 (95% CI -378.1937 - 1711.9553)	0.380124
Glycerophospholipids lysoPC.a.C28.1	459.8121 (95% CI -106.597 - 1026.2212)	475.3304 (95% CI -172.8129 - 1123.4737)	0.955654
Glycerophospholipids PC.aa.C28.1	1802.725 (95% CI 1693.8054 - 1911.6446)	1811.9014 (95% CI 1677.0125 - 1946.7903)	0.92576
Glycerophospholipids PC.aa.C30.0	2564.1125 (95% CI 2361.748 - 2766.477)	2573.9577 (95% CI 2346.7416 - 2801.1738)	0.970562
Glycerophospholipids PC.aa.C32.0	10164.8 (95% CI 9466.136 - 10863.464)	9771.2113 (95% CI 9084.5633 - 10457.8593)	0.416248
Glycerophospholipids PC.aa.C32.1	10617.8375 (95% CI 9270.8748 - 11964.8002)	9894.9155 (95% CI 8754.8834 - 11034.9476)	0.421334
Glycerophospholipids PC.aa.C32.2	2545.1125 (95% CI 2247.2752 - 2842.9498)	2368.3239 (95% CI 2122.4897 - 2614.1581)	0.345754
Glycerophospholipids PC.aa.C32.3	260.3975 (95% CI 243.383 - 277.412)	257.5493 (95% CI 237.0508 - 278.0478)	0.810979
Glycerophospholipids PC.aa.C34.1	170534.225 (95% CI 158650.3246 - 182418.1254)	158411.831 (95% CI 147075.2959 - 169748.3661)	0.148277
Glycerophospholipids PC.aa.C34.2	254021.725 (95% CI 238074.3688 - 269969.0812)	236396.1408 (95% CI 218825.0343 - 253967.2473)	0.128968
Glycerophospholipids PC.aa.C34.3	10241.6375 (95% CI 9478.2352 - 11005.0398)	9698.507 (95% CI 8783.0057 - 10614.0083)	0.359569
Glycerophospholipids PC.aa.C34.4	1063.0125 (95% CI 970.0927 - 1155.9323)	983.3803 (95% CI 904.5878 - 1062.1728)	0.192577
Glycerophospholipids PC.aa.C36.0	2406.5395 (95% CI 2039.7807 - 2773.2983)	2461.6812 (95% CI 1625.1668 - 3298.1956)	0.929583
Glycerophospholipids PC.aa.C36.1	42879.1125 (95% CI 40399.2156 - 45359.0094)	42165.6479 (95% CI 39188.9585 - 45142.3373)	0.706492
Glycerophospholipids PC.aa.C36.2	185946.1375 (95% CI 174650.7527 - 197241.5223)	179501.8286 (95% CI 167021.754 - 191981.9032)	0.425394
Glycerophospholipids PC.aa.C36.3	90408.075 (95% CI 85616.6439 - 95199.5061)	87346.9437 (95% CI 81957.7278 - 92736.1596)	0.386101
Glycerophospholipids PC.aa.C36.5	25307.925 (95% CI 22099.7639 - 28516.0861)	22105.6714 (95% CI 19291.0737 - 24920.2691)	0.147414
Glycerophospholipids PC.aa.C36.6	797.4875 (95% CI 719.2359 - 875.7391)	701.7183 (95% CI 630.2294 - 773.2072)	0.076595
Glycerophospholipids PC.aa.C38.0	2732.0625 (95% CI 2564.4804 - 2899.6446)	2572.3239 (95% CI 2382.8145 - 2761.8333)	0.206856
Glycerophospholipids PC.aa.C38.1	1145.7375 (95% CI 1043.2509 - 1248.2241)	1201.5882 (95% CI 889.3812 - 1513.7952)	0.722978
Glycerophospholipids PC.aa.C38.3	38180.6125 (95% CI 35895.9622 - 40465.2628)	37374.8571 (95% CI 34942.281 - 39807.4332)	0.628198
Glycerophospholipids PC.aa.C38.4	93945.15 (95% CI 87855.9105 - 100034.3895)	86085.5143 (95% CI 80338.0398 - 91832.9888)	0.068258
Glycerophospholipids PC.aa.C40.1	401.0556 (95% CI 333.5327 - 468.5786)	549.8 (95% CI 241.6704 - 857.9296)	0.260205
Glycerophospholipids PC.aa.C40.2	271.61 (95% CI 244.7426 - 298.4774)	247.6394 (95% CI 207.4832 - 287.7956)	0.31349
Glycerophospholipids	572.6125	522.8592	0.353426

PC.aa.C40.3	(95% CI 495.2151 - 650.0099)	(95% CI 449.2072 - 596.5112)	
Glycerophospholipids PC.aa.C40.4	2242.1875 (95% CI 2076.1279 - 2408.2471)	2206.9577 (95% CI 2048.2704 - 2365.645)	0.757802
Glycerophospholipids PC.aa.C40.5	8391.5875 (95% CI 7824.5597 - 8958.6153)	7985.6479 (95% CI 7337.11 - 8634.1858)	0.353549
Glycerophospholipids PC.aa.C42.0	474.1625 (95% CI 439.7747 - 508.5503)	500.9296 (95% CI 452.8699 - 548.9893)	0.366453
Glycerophospholipids PC.aa.C42.1	228.8725 (95% CI 185.8176 - 271.9274)	221.6761 (95% CI 194.1808 - 249.1714)	0.779071
Glycerophospholipids PC.aa.C42.2	196.2785 (95% CI 181.4416 - 211.1154)	194.2225 (95% CI 136.1693 - 252.2757)	0.932799
Glycerophospholipids PC.aa.C42.4	136.6225 (95% CI 127.9267 - 145.3183)	135.3114 (95% CI 114.9831 - 155.6397)	0.892175
Glycerophospholipids PC.aa.C42.5	337.3875 (95% CI 308.2558 - 366.5192)	321 (95% CI 281.776 - 360.224)	0.493012
Glycerophospholipids PC.aa.C42.6	445.275 (95% CI 338.5389 - 552.0111)	403.8592 (95% CI 335.1419 - 472.5765)	0.526649
Glycerophospholipids PC.ae.C30.2	64.8688 (95% CI 53.803 - 75.9346)	67.6423 (95% CI 53.7225 - 81.5621)	0.766774
Glycerophospholipids PC.ae.C32.1	1962.2875 (95% CI 1788.0148 - 2136.5602)	1941.2143 (95% CI 1794.7755 - 2087.6531)	0.842423
Glycerophospholipids PC.ae.C32.2	496.75 (95% CI 459.39 - 534.11)	458.5493 (95% CI 427.6599 - 489.4387)	0.118968
Glycerophospholipids PC.ae.C34.1	6247.075 (95% CI 5902.1273 - 6592.0227)	6461.7606 (95% CI 6027.2765 - 6896.2447)	0.450872
Glycerophospholipids PC.ae.C34.2	7659.7125 (95% CI 7150.7521 - 8168.6729)	8234.493 (95% CI 7609.7149 - 8859.2711)	0.156834
Glycerophospholipids PC.ae.C34.3	5057.775 (95% CI 4670.2545 - 5445.2955)	5134.3944 (95% CI 4677.1182 - 5591.6706)	0.813931
Glycerophospholipids PC.ae.C36.0	590.5125 (95% CI 546.4987 - 634.5263)	605.662 (95% CI 495.3703 - 715.9537)	0.803479
Glycerophospholipids PC.ae.C36.1	5110.2 (95% CI 4705.5326 - 5514.8674)	5060.1429 (95% CI 4697.9736 - 5422.3122)	0.847816
Glycerophospholipids PC.ae.C36.2	8481.625 (95% CI 7812.6769 - 9150.5731)	8715.8571 (95% CI 8058.1548 - 9373.5594)	0.634967
Glycerophospholipids PC.ae.C36.3	4989.775 (95% CI 4643.6637 - 5335.8863)	5516.3521 (95% CI 5079.1649 - 5953.5393)	0.060857
Glycerophospholipids PC.ae.C36.4	15137.6125 (95% CI 14096.7031 - 16178.5219)	15786.3714 (95% CI 14657.0129 - 16915.7299)	0.410425
Glycerophospholipids PC.ae.C36.5	10000.1 (95% CI 9360.9453 - 10639.2547)	9352.5352 (95% CI 8694.2888 - 10010.7816)	0.163502
Glycerophospholipids PC.ae.C38.1	363.6683 (95% CI 83.7939 - 643.5427)	253.46 (95% CI 153.9858 - 352.9342)	0.472775
Glycerophospholipids PC.ae.C38.2	1071.1875 (95% CI 876.5941 - 1265.7809)	1155.7887 (95% CI 894.9811 - 1416.5963)	0.613557
Glycerophospholipids PC.ae.C38.3	2393.1625 (95% CI 2134.4965 - 2651.8285)	2457.1549 (95% CI 2297.2408 - 2617.069)	0.698342
Glycerophospholipids PC.ae.C38.4	9345.9875 (95% CI 8824.3929 - 9867.5821)	10152.7324 (95% CI 9501.8143 - 10803.6505)	0.056673
Glycerophospholipids PC.ae.C38.5	14023.25 (95% CI 13208.0733 - 14838.426)	13881.6197 (95% CI 13029.6974 - 14733.542)	0.793575
Glycerophospholipids PC.ae.C38.6	6516.375 (95% CI 6125.4466 - 6907.3034)	6244.8169 (95% CI 5829.9827 - 6659.6511)	0.33361
Glycerophospholipids PC.ae.C40.1	1028.8125 (95% CI 932.2061 - 1125.4189)	919.5143 (95% CI 859.1746 - 979.854)	0.063922
Glycerophospholipids PC.ae.C40.2	1353.775 (95% CI 1216.7212 - 1490.8288)	1421.4789 (95% CI 1260.8924 - 1582.0654)	0.530456

Glycerophospholipids PC.ae.C40.3	572.2 (95% CI 546.1666 - 598.2334)	626.7746 (95% CI 566.4923 - 687.0569)	0.091534
Glycerophospholipids PC.ae.C40.4	1379.55 (95% CI 1304.7507 - 1454.3493)	1485.7887 (95% CI 1396.2462 - 1575.3312)	0.073128
Glycerophospholipids PC.ae.C40.5	2369.5375 (95% CI 2260.2096 - 2478.8654)	2469.8169 (95% CI 2293.3157 - 2646.3181)	0.334997
Glycerophospholipids PC.ae.C40.6	3527.9625 (95% CI 3319.9962 - 3735.9288)	3464.5352 (95% CI 3194.9875 - 3734.0829)	0.702044
Glycerophospholipids PC.ae.C42.0	391.1786 (95% CI 309.1357 - 473.2215)	413.725 (95% CI 282.6848 - 544.7652)	0.795569
Glycerophospholipids PC.ae.C42.1	266.08 (95% CI 249.7442 - 282.4158)	253.4429 (95% CI 225.0806 - 281.8052)	0.421277
Glycerophospholipids PC.ae.C42.2	453.6125 (95% CI 396.5162 - 510.7088)	444.1972 (95% CI 405.9513 - 482.4431)	0.773234
Glycerophospholipids PC.ae.C42.3	584.1875 (95% CI 499.6528 - 668.7222)	549.6479 (95% CI 496.6083 - 602.6875)	0.489653
Glycerophospholipids PC.ae.C42.4	549.1282 (95% CI 504.4966 - 593.7598)	603.5294 (95% CI 559.8266 - 647.2322)	0.092739
Glycerophospholipids PC.ae.C42.5	1509.2375 (95% CI 1398.6432 - 1619.8318)	1566.2113 (95% CI 1471.2923 - 1661.1303)	0.452173
Glycerophospholipids PC.ae.C44.3	74.4938 (95% CI 69.7341 - 79.2535)	113.8971 (95% CI 59.9049 - 167.8893)	0.128614
Glycerophospholipids PC.ae.C44.4	353.325 (95% CI 259.429 - 447.221)	343.338 (95% CI 315.0028 - 371.6732)	0.84461
Glycerophospholipids PC.ae.C44.5	1350.15 (95% CI 1259.1496 - 1441.1504)	1426.5143 (95% CI 1322.3885 - 1530.6401)	0.275664
Glycerophospholipids PC.ae.C44.6	904.3875 (95% CI 834.4996 - 974.2754)	948.5493 (95% CI 861.4148 - 1035.6838)	0.436031
Sphingolipids SM.C16.0	100737.6375 (95% CI 95679.9984 - 105795.2766)	99224.338 (95% CI 92138.5688 - 106310.1072)	0.714396
Sphingolipids SM.C16.1	14226.7 95% CI 13425.0443 - 15028.3557)	13382.7042 (95% CI 12460.2143 - 14305.1941)	0.162311
Sphingolipids SM.C18.0	24655.65 (95% CI 22811.1951 - 26500.1049)	23006.4366 (95% CI 20958.1333 - 25054.7399)	0.234845
Sphingolipids SM.C18.1	11336.5375 (95% CI 10635.1092 - 12037.9658)	10500.7042 (95% CI 9722.6177 - 11278.7907)	0.111441
Sphingolipids SM.C20.2	275.6228 (95% CI 248.6863 - 302.5593)	289.9314 (95% CI 200.7187 - 379.1441)	0.759218
Sphingolipids SM.C24.0	20245.7625 (95% CI 17585.9426 - 22905.5824)	20607.169 (95% CI 17096.9232 - 24117.4148)	0.883505
Sphingolipids SM.C26.0	137.3138 (95% CI 77.5855 - 197.0421)	142.4141 (95% CI 95.5245 - 189.3037)	0.904056
Sphingolipids SM.C26.1	390.7625 (95% CI 348.9633 - 432.5617)	452.0563 (95% CI 290.9744 - 613.1382)	0.450355
Sphingolipids SM.OH.C14.1	4600.2625 (95% CI 4054.6319 - 5145.8931)	4680.0563 (95% CI 4247.0782 - 5113.0344)	0.833738
Sphingolipids SM.OH.C16.1	2664.7625 (95% CI 2454.5316 - 2874.9934)	3049.3239 (95% CI 2456.0848 - 3642.563)	0.211839
Sphingolipids SM.OH.C22.1	10615.35 (95% CI 10030.0392 - 11200.6608)	11791.5775 (95% CI 10230.7314 - 13352.4236)	0.150316
Sphingolipids SM.OH.C22.2	10787.3625 (95% CI 10202.2733 - 11372.4517)	10692.7183 (95% CI 9736.7359 - 11648.7007)	0.845789
Sphingolipids SM.OH.C24.1	1147.275 (95% CI 983.7129 - 1310.8371)	1266.1127 (95% CI 1015.2182 - 1517.0072)	0.431075

CI - confidence interval. Mean values together with the 95% confidence intervals of the means are presented. 96
